# Structural insights into positive and negative allosteric regulation of a G protein-coupled receptor through protein-lipid interactions

**DOI:** 10.1038/s41598-018-22735-6

**Published:** 2018-03-13

**Authors:** Agustín Bruzzese, Carles Gil, James A. R. Dalton, Jesús Giraldo

**Affiliations:** 1 Laboratory of Molecular Neuropharmacology and Bioinformatics, Institut de Neurociències and Unitat de Bioestadística, Universitat Autònoma de Barcelona, 08193 Bellaterra, Spain; 2 Network Biomedical Research Centre on Mental Health (CIBERSAM), 08193 Bellaterra, Spain; 3 Department of Biochemistry and Molecular Biology, Institut de Neurociències, Universitat Autònoma de Barcelona, 08193 Bellaterra, Spain

## Abstract

Lipids are becoming known as essential allosteric modulators of G protein-coupled receptor (GPCRs). However, how they exert their effects on GPCR conformation at the atomic level is still unclear. In light of recent experimental data, we have performed several long-timescale molecular dynamics (MD) simulations, totalling 24 μs, to rigorously map allosteric modulation and conformational changes in the β_2_ adrenergic receptor (β2AR) that occur as a result of interactions with three different phospholipids. In particular, we identify different sequential mechanisms behind receptor activation and deactivation, respectively, mediated by specific lipid interactions with key receptor regions. We show that net negatively charged lipids stabilize an active-like state of β2AR that is able to dock G_s_α protein. Clustering of anionic lipids around the receptor with local distortion of membrane thickness is also apparent. On the other hand, net-neutral zwitterionic lipids inactivate the receptor, generating either fully inactive or intermediate states, with kinetics depending on lipid headgroup charge distribution and hydrophobicity. These chemical differences alter membrane thickness and density, which differentially destabilize the β2AR active state through lateral compression effects.

## Introduction

G protein-coupled receptors (GPCRs) are implicated in the regulation of many physiological and pathological processes^1^. For this reason, GPCRs are a major target of current marketed drugs^2^. GPCRs are seven-helix transmembrane domain (7TM) receptors that reside in and signal through the lipid membrane of the cell^1^. From an atomistic perspective, GPCRs are flexible proteins that fluctuate between several conformations that can be broadly grouped into inactive, active and intermediate states, and which can be modulated by ligands^3^. The transitions between different conformations, for instance from active to inactive state, can occur along time scales of nanoseconds to milliseconds^4,5^. However, it is still unclear how exactly ligand binding induces (or selects for) receptor activation and elicits efficient signal transduction^3,4,6,7^. In addition to modulation by ligands, phospholipids have been shown to alter the activity of certain proteins by interacting with transmembrane helices, for example in sarcoplasmic reticulum Ca^2+^-ATPase, large-conductance mechanosensitive channel MscL, multidrug transporter LmrP, and rhodopsin^8,9^. These effects have typically been shown to be mediated by H-bond formation (or lack thereof) between the protein and phospholipid headgroups^8^. In addition, interactions with cholesterol have been shown to be important for GPCRs, e.g. influencing ionic-lock formation in A2A adenosine receptor^10^ and thermal stability of β_2_ adrenergic receptor (β2AR)^11^.

Molecular dynamics (MD) is a suitable computational technique for studying GPCR flexibility in its membrane environment. MD simulations at atomic resolution can give information on specific molecular processes, including interactions of proteins with lipids, receptor-ligand binding, and receptor conformational change^12^. Recent X-ray crystal structures of β2AR have provided high-resolution insights into two major conformations associated with GPCR function: an inactive inverse agonist-bound state^13^ and an active state in complex with an agonist and G_s_ protein^14^. These crystal structures constitute a reference point for comparison between active and inactive states (see SI Figs 1 and 2). Moreover, the β2AR is widely expressed throughout the body and can adopt highly diverse conformations with each of them having particular signalling patterns^15^. As such, this receptor constitutes an ideal paradigmatic system for the structural exploration of its (in)activation, both by theoretical and experimental approaches^4,16–19^. From a structure-function perspective, by comparing the active and inactive crystal structures, some authors have emphasized the importance of the rearrangement of transmembrane (TM) helices 5, 6 and 7 in transmitting the signal through the membrane^3–5,20,21^. Further research in this area may include the mechanisms underlying the transitions and fluctuations of β2AR amongst its many conformational states.

Several MD simulation studies of β2AR, initialized from its various crystal structures, have been performed before e.g.^4,5,15,16,18,22–33^. Although precise methodological protocols differ, most of these MD simulations share a common theme. By way of summary, the majority do not include intracellular loop 3 (ICL3) of β2AR^4,5,16,18,22,26–28,30–33^, which is missing in β2AR crystal structures^11,13,14,34–41^ and therefore requires explicit modelling. When the active receptor state(s) is simulated, co-crystallized molecules such as G_s_ protein or nanobody are usually first removed^4,22,29,31,32^. In addition, most simulations of β2AR are performed within a homogeneous membrane consisting of 1-palmitoyl-2-oleoyl-sn-glycero-3-phosphocholine (POPC) lipids^4,5,16,18,22–28,30–33^. This is due to the relative abundance of this particular phospholipid in healthy mammalian membranes^42^. Noteworthy, MD simulations of β2AR carried out from the inactive state crystal structure suggest that within this state exist two conformations that are in equilibrium: an inactive (broken ionic-lock) and a very-inactive (closed ionic-lock)^15^. On the other hand, MD simulations starting from the active crystal structure in a POPC membrane (without bound G protein or nanobody) usually result in the spontaneous deactivation of the receptor, even with an agonist bound^4^. This suggests that the preferred state of β2AR under normal conditions is the inactive, with TM6 fluctuating between a set of inward orientations (representing very inactive, inactive or intermediate), and only adopting an active state with an outward TM6 when a G protein is bound^3,5,17^. However, these observations may be influenced by the absence of ICL3 (which connects TM5 and TM6) in some of these MD simulations. Indeed, in recent MD studies which did include ICL3, receptor behaviour was found to be altered because of noticeable allosteric effects^23,25^. Likewise, experimental data suggests ICL3 is important for the spontaneous activation of β2AR^43^. Interestingly, recent data gathered in a β2AR ligand binding study suggests that protein and membrane interplay improves the interaction between protein and ligand and could be important for drug development^26^.

**Figure 1:**
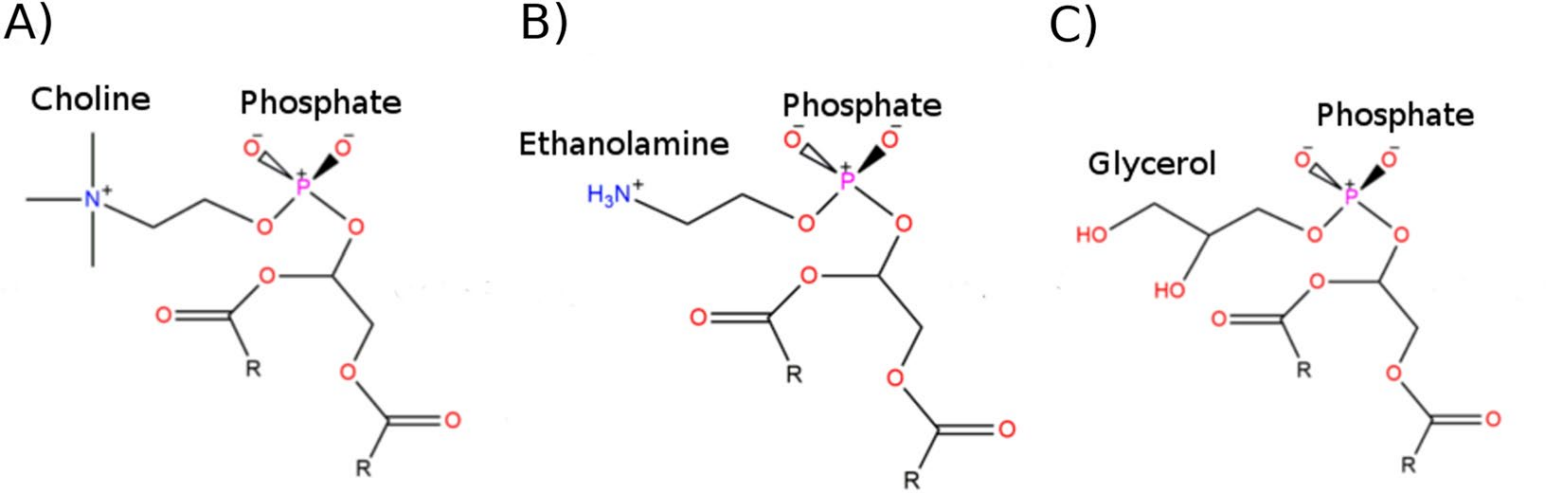
Structural comparison of DOPC, DOPE and DOPG unsaturated lipids. DOPC (**A**) contains two fatty-acid chains of 18 carbons each (R) and its headgroup, which is composed of a positively-charged choline group bound to the phosphate group. DOPE (**B**) contains identical fatty-acid chains but its headgroup is composed of a positively-charged ethanolamine group bound to the phosphate group. DOPG (**C**) contains identical fatty-acid chains but its headgroup is composed of a neutral glycerol group bound to the phosphate group.

In a related experimental study using artificial nanodiscs, the phospholipids 1,2-dioleoyl-sn-glycero-3-phosphoglycerol (DOPG) and 1,2-dioleoyl-sn-glycero-3-phospho-ethanolamine (DOPE) were shown to be strong allosteric modulators of β2AR, stabilizing and destabilizing the active receptor state, respectively^44^. Furthermore, 1,2-dioleoyl-sn-glycero-3-phosphocholine (DOPC), a close relative of POPC, does not favour either active or inactive states and instead allows the receptor to explore different conformations without particular preference^44^. The differences between these three phospholipids lie in the electrostatic charges of their headgroups as all contain the same unsaturated fatty acid chains (two chains of 18 carbons each). Both DOPC (Fig. 1A) and DOPE (Fig. 1B) contain a positively charged headgroup which, in combination with the negatively charged phosphate group, yields a dipole with net neutral charge. The main difference between them is that DOPC has three methyl groups connected to its headgroup nitrogen atom while DOPE has three hydrogens. This makes the DOPC headgroup more hydrophobic, while the corresponding hydrogens in DOPE are free to make ionic/H-bond interactions with other molecules. Other authors have suggested that DOPE and DOPC have different orientations in the membrane due to different intermolecular interactions between P-N groups^45–47^. Unlike the other two, DOPG (Fig. 1C) has a neutral headgroup with two polar hydroxyls which, in combination with the negatively charged phosphate group, yields a net negatively charged phospholipid.

Lipid characterizations of human tissue show that dioleoyl (DO) fatty acid chains are abundant in membranes^48^. Although DOPG lipids specifically have low biological expression (<2%^44^), it represents over >7% of all β2AR-bound phospholipids (>16% for PG lipids in general)^44^, which possibly indicates enrichment in the vicinity of β2AR. As lipid composition can be affected by disease, as shown by membrane changes in the brains of Alzheimer’s and Parkinson’s patients^49–52^, these effects could have a bearing on the activity of GPCRs such as β2AR. Along this line of research, recently published MD simulations of β2AR inserted in a homogenous 1-palmitoyl-2-oleoyl-sn-glycero-3-phosphoglycerol (POPG) membrane has shown that a single anionic lipid is able to disrupt the receptor ionic lock^16^, a typical feature of the inactive (or very-inactive) receptor state^15,16^. This occurs through formation of a salt-bridge between the lipid phosphate group and residue R^3.50^ (Ballesteros-Weinstein numbering^53^) of the ionic-lock^16^. Thus, it suggests a possible mechanism by which net negatively charged phospholipids can potentially interact with positively charged residues located at the intracellular side of β2AR, thereby possibly contributing to the (de)stabilization of the (in)active receptor state.

Despite these findings, a structural explanation of both positive and negative allosteric regulation by phospholipids on β2AR activity remains unclear. To this aim, we focus on the allosteric effects of DOPG, DOPE, and DOPC phospholipids on the conformational changes of the active state of β2AR, including modelled ICL3, in unbiased atomistic long-timescale MD simulations. Specifically, we investigate from a structural point-of-view whether these different phospholipids can allosterically modulate β2AR in a reproducible fashion, either positively or negatively as suggested in analogous experiments^44^, by acting on specific intracellular residues. From a more general perspective, our results potentially open new insights into how phospholipids might influence GPCR behaviour and signalling at a molecular level. In addition, the present study suggests that the choice of membrane lipid composition in computational MD simulations of GPCRs can significantly affect outcome.

## Materials and Methods

### β2AR modelling

The crystal structure of the active state of β2AR (PDB entry: 3SN6)^14^ was selected. The G_s_ protein complex, nanobody, lysozyme-fusion protein, and co-crystallized agonist were removed from the structure. Mutated residues (T96, T98 and E187) were substituted by native ones (M96, M98 and N187) in order to obtain a *wt* receptor sequence. The crystallographic-missing extracellular loop 2 (ECL2) was completed by homology modelling to the corresponding region of the inactive crystal structure of β2AR (PDB entry: 2RH1)^13^ using MODELLER v9.14^54^. Intracellular loop 3 (ICL3) was confirmed using secondary structure prediction tools, Jpred^55^ and PSIpred^56^ (residues 238 to 262) and then modelled using MODELLER. The complete receptor was then energy-minimized in the AMBER-14SB force-field^57^ with CHIMERA^58^.

### Molecular dynamics (MD) simulations

Three all-atom MD systems were constructed, consisting of β2AR in active state (as described above) in three homogeneous membranes: DOPG, DOPE and DOPC (Fig. 1), respectively, using CHARMM-GUI^59^. Each receptor-membrane system was solvated with TIP3P water molecules above and below the membrane, with a concentration of 0.3 M KCl for zero system net charge. On the protein, six residues were protonated according to previously published MD simulation protocols specific for β2AR, as well as being consistent with protocols used for other homologous Class A GPCRs^4,22,60^: D79^2.50^, E122^3.41^, D130^3.49^, D234^5.73^, E237^5.76^ and E268^6.30^ (Ballesteros-Weinstein numbering^53^ as superscript). MD simulations were performed using the CHARMM36 force field^61^ and ACEMD software^62^. Briefly, each receptor-membrane system was equilibrated for 28 ns at 300 K and 1 atmosphere. During the initial 8 ns of equilibration, the protein was harmonically restrained and progressively released over 2 ns steps. During the final 20 ns of equilibration, no restraints were applied. For each receptor-membrane system a production run of 4 μs was performed without restraints under the same conditions, with a second replicate simulation executed in each case to verify observations. This constitutes a total production simulation time of 24 μs.

### MD simulation analysis

For each MD simulation trajectory, root mean square deviation (RMSD) measurements of the transmembrane (TM) domain (TM helices 1–7 plus helix 8), as well as TM6 or ICL3 by itself, were made with VMD software^63^ v1.9.2 in order to evaluate protein conformational changes and stability of receptor state. The distance between the Cα atoms of ionic-lock residues R131^3.50^ on TM3 and E268^6.30^ on TM6 was used as an indication of proximity between these two helices. RMSD of the “triad core”, named by Huang *et al*.^64^, (all heavy atoms of residues: I121^3.40^, P211^5.50^ and F282^6.44^) was used as an indicator of receptor state^4,65^. In order to assess membrane characteristics and protein-membrane interactions: i) the distance(s) between ionic-lock residue R131^3.50^ (terminal nitrogen atoms) and closest lipid phosphate group (centre-of-mass) was measured with PLUMED^66^; (ii) membrane thickness calculated as average distance from lower to upper-leaflet phosphorus atoms, implementing default settings of MEMBPLUGIN^67^ within VMD^63^ over total simulation time; (iii) membrane density calculated across bilayer Z-axis using default settings of MEMBPLUGIN^67^, analyzing mean mass of fatty acid chains and phosphate groups across whole membrane over total simulation time; (iv) area per lipid calculated by dividing total membrane area by total number of upper leaflet lipids (membrane area defined by its maximum X,Y dimensions minus cross-sectional area of protein in the same plane); (v) number of protein-lipid electrostatic interactions calculated every 4 ns between charged lipid atoms of inner-leaflet and charged protein TM6 atoms (counting all charged atom-atom pairs with distance <4.5 Å) using a custom-made script executed within VMD^63^; (vi) radial distribution function implemented twice with the Radial Pair Distribution plugin^68^ within VMD^63^: firstly, *g(r)* of whole TM6 with respect to lower-leaflet lipid phosphate groups and secondly, *g(r)* of positively charged atoms of four lysine sidechains on intracellular side of TM6 (K263^6.25^, K267^6.29^, K270^6.32^, K273^6.35^, Ballesteros-Weinstein numbering^53^ as superscript) and negatively charged oxygen atoms of lower-leaflet lipid phosphate groups. As a further validation of receptor state, the co-crystallized G_s_α protein (PDB entry: 3SN6) was docked to the intracellular side of relevant MD-generated β2AR structures (as well as re-docked into the original active β2AR crystal structure, PDB entry: 3SN6, as a control). The Rosetta online server (ROSIE) was used for protein-protein docking^69^ with the following protocol: (i) receptor conformation taken from the end of its respective 4 μs MD simulation and superimposed over the active crystal structure of β2AR containing its G_s_α protein (PDB entry: 3SN6)^14^ (ii) the original crystallized receptor removed from the complex, (iii) the structure of G_s_α moved 3 Å away from the MD-generated receptor structure so that there are no steric clashes and clear space is apparent between both proteins, (iv) protein-protein docking is initiated. As ICL3 is long and potentially highly flexible, it was removed prior to protein-protein docking (during step iii) so as not to create steric conflicts with G_s_α during docking (ROSIE is not able to move backbone of loops during docking). However, all other loops and receptor structural elements were maintained. All analytical plots were generated using GNUplot^70^ version 4.449.

## Results

β2AR is one of the most studied and frequently crystallized Class A GPCRs. However, the structure of intracellular loop 3 (ICL3) that connects TM5 and TM6 is unknown because it is missing in all β2AR crystal structures. ICL3 has recently been shown to be potentially important in β2AR activity^23,25^. On this basis, we *ab initio* modelled ICL3 prior to performing MD simulations. As a result, β2AR contains 13 positively charged residues located at the intracellular ends of TM5, TM6, and ICL3 (SI Fig. 1), which have been suggested as important for G protein interaction^71^. β2AR has been crystallized in both active (G_s_ protein-bound, PDB entry: 3SN6)^14^ and inactive (carazolol-bound, PDB entry: 2RH1)^13^ states. Briefly, one of the most remarkable differences between them is the orientation of TM6. In the active state, an outward movement of ~14 Å is observed (SI Fig. 2). As a result, the ionic-lock (R^3.50^ and E^6.30^, with Ballesteros-Weinstein numbering^53^ as superscript, which indicates relative residue position along each TM helix) reaches a distance of 18.9 Å in the active state but only 11.1 Å in the inactive (SI Fig. 2) ^13,14^. Other features of the active state include a shortening of TM7 (below the conserved NPxxY motif^4^) where it connects with H8, and an ordered helical second intracellular loop (ICL2), which is disordered in the inactive state (SI Fig. 2)^13^. Mechanistically, the motion of three residues in the core of β2AR has been shown to be important in the receptor (de)activation process. These residues constitute the triad core: I^3.40^, P^5.50^ and F^6.44^ (Ballesteros-Weinstein numbering^53^ as superscript), with its packing rearrangement (SI Fig. 2) involved in the relative motions of TM3, TM5 and TM6, serving as a connector between extracellular and intracellular receptor regions^4,65^. In light of observations of relevant β2AR crystal structures, proposed ICL3 functionality^23^, and experimental data that shows β2AR is strongly allosterically modulated by different phospholipids^44^, we have investigated the sum of these effects by employing long-timescale MD simulations of the active state of apo β2AR (PDB entry: 3SN6)^14^ in three different homogenous membranes consisting of lipids: DOPC, DOPE and DOPG, respectively. In order to quantify the respective modulation of receptor state by each lipid, six metrics were utilised: conformational re-arrangement of the triad core, ionic-lock distance changes, RMSD of total protein, ICL3, and TM6, as well as radial distribution *g(r)* of TM6 with respect to lipids.

### Lipid DOPC partially inactivates β2-adrenergic receptor

**Figure 2:**
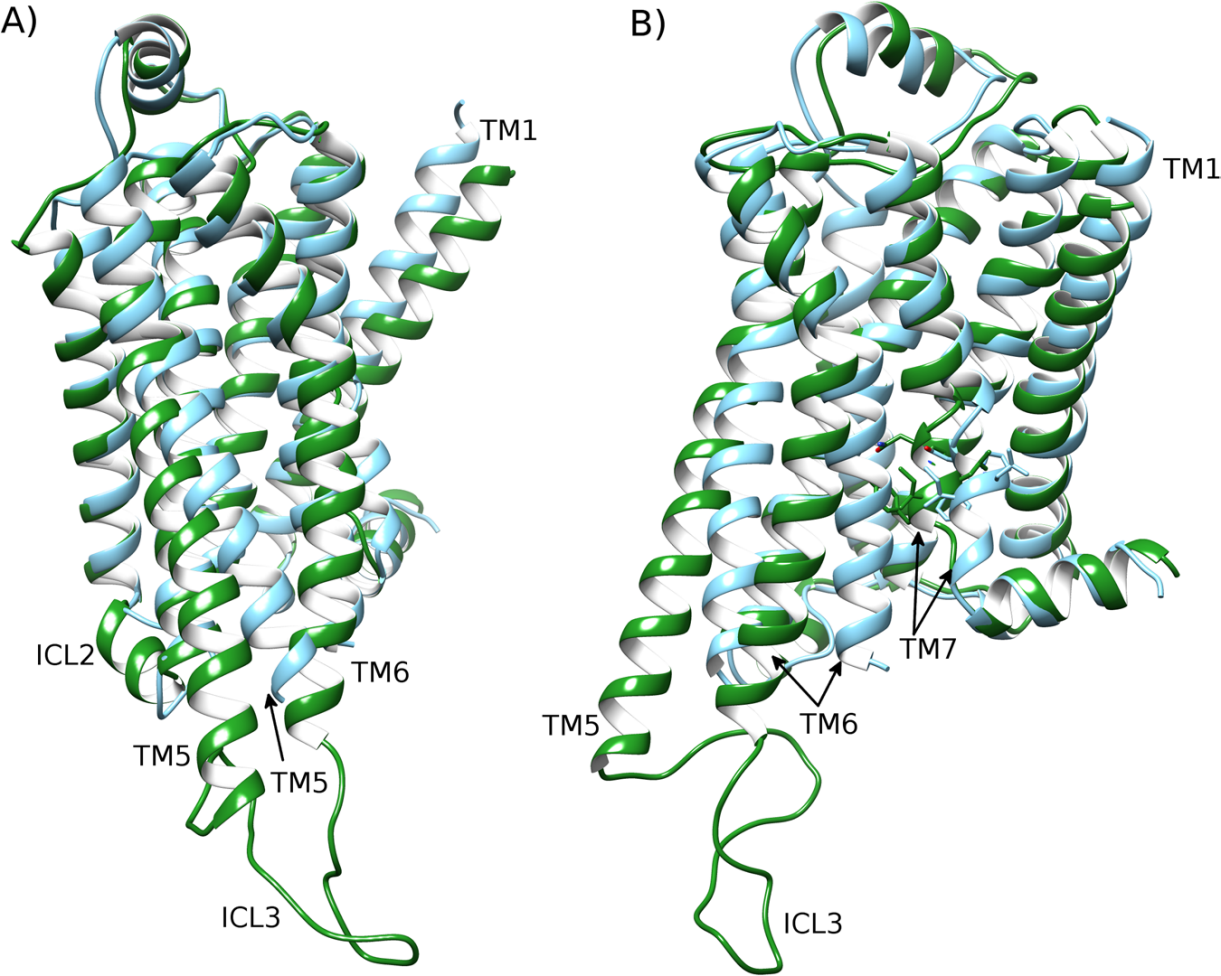
Partial inactivation of β_2_AR in a DOPC membrane. Superposition of the inactive-state crystal structure of β_2_AR (PDB id: 2RH1, blue) and an MD-generated conformation achieved after 4 μs within a DOPC membrane (green), showing a 90° rotation between (**A**) and (**B**) around the membrane plane. At the beginning of the MD simulation, β_2_AR starts in its active crystal state (PDB id: 3SN6). Intracellular loops (ICL) 2 and 3, and transmembrane (TM) helices 5, 6 and 7 are labelled. Residues of the NPxxY motif on TM7 are displayed.

**Figure 3:**
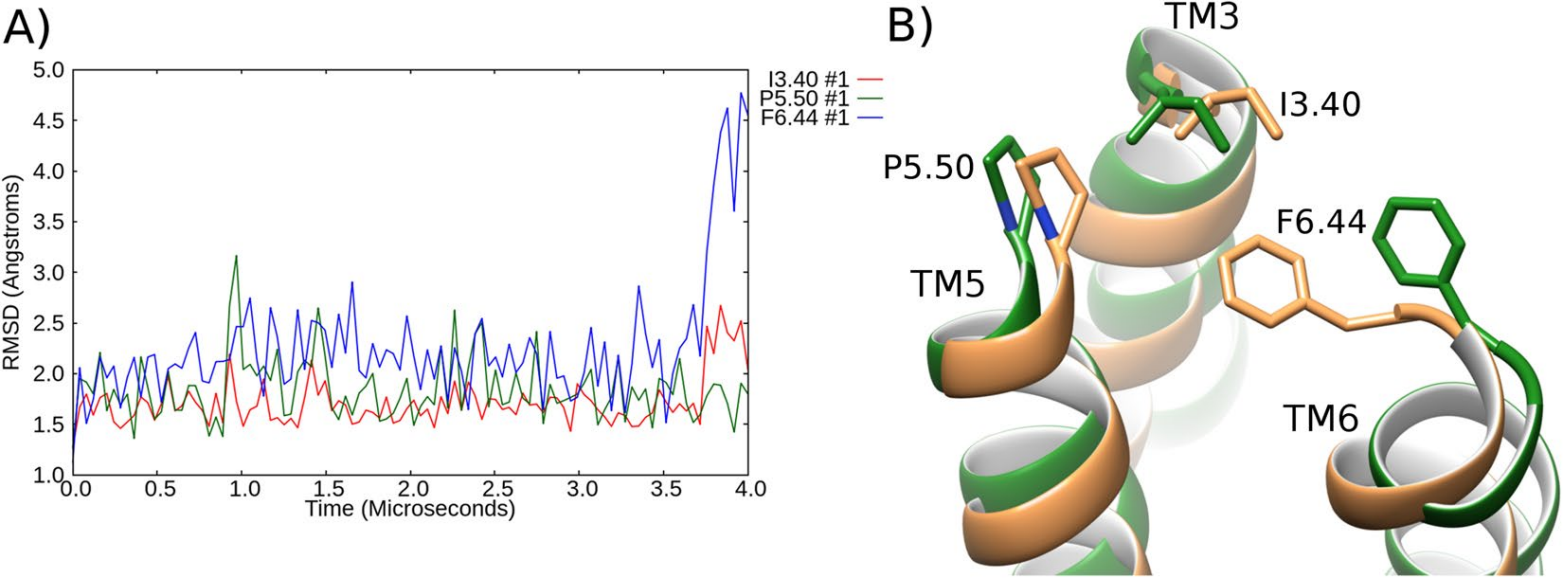
Partial inactivation of the triad core of β2AR in a DOPC membrane. (**A**) RMSD profile of the conformational changes of the triad core: I^3.40^ (red), P^5.50^ (green) and F^6.44^ (blue) over a 4 μs MD simulation in a DOPC membrane, compared to the active crystal structure (PDB id: 3SN6). (**B**) Superposition of the triad core observed at 4 μs compared to the active crystal structure triad core (orange, PDB id: 3SN6). Relevant residues are labelled: proline (P), isoleucine (I), phenylalanine (F) on transmembrane (TM) helices 3, 5, 6.

In a 4 μs MD simulation of β2AR in a homogenous membrane of DOPC, ICL3 shows high flexibility and transiently adopts different conformations during the first microsecond (SI Fig. 3). During this time, the loop does not interact with the membrane but gradually moves to an inward position, interacting with ICL2 on the intracellular side of the receptor, as well as intermittently with H8 (SI Fig. 3). From 1 μs onwards, β2AR maintains ICL3 in this inward conformation (SI Fig. 3). The conformational changes of ICL3 precede a change in the conformation of TM6, which occurs from 2 μs onwards. In an RMSD profile of TM6 with respect to the inactive-state crystal structure of β2AR (PDB entry: 2RH1), TM6 begins at 7.0 Å but decreases to 3.5–5.5 Å from 2 μs onwards, finishing at 4.7 Å (SI Fig. 4). This suggests that the receptor is gradually being deactivated (closer in conformation to the inactive state). However, over 4 μs, TM6 does not fully reach the conformation of the inactive crystal structure (PDB id: 2RH1) and ICL2 remains in its helical state (Fig. 2). This is despite the receptor containing certain features of the inactive crystal structure such as helical extension at the intracellular end of TM7 (Fig. 2). Likewise, conformational changes are also observed in the triad core, which maintains an active-like conformation of I^3.40^, P^5.50^ and F^6.44^ until 3.5 μs (Fig. 3), at which point, the RMSD of F^6.44^ increases to 4.6 Å, which resembles an inactive-like conformation. Supporting the partial inactivation of β2AR into an intermediate state, the ionic-lock distance gradually decreases to 11.1 Å over 4 μs (SI Fig. 5) and the TM domain reaches an RMSD of 3.4 Å compared to the inactive crystal structure (SI Fig. 6). In general terms, this tallies with the experimental results of Dawaliby *et al*.^44^, which show that DOPC neither favours the active or inactive state of β2AR, but in effect here weakly destabilises the active^44^. Likewise, these results are supported by the computational results of Dror *et al*.^4^ whose long-timescale MD simulations of β2AR in a POPC membrane also exhibited gradual inactivation. Although POPC is not the same as DOPC (POPC contains fatty-acid chains of 18 and 16 carbons, respectively), they both contain the same lipid headgroup and therefore at a chemical level the inter-lipid, water-lipid and protein-lipid interactions would be expected to be similar, which allows for comparison.

### Lipid DOPE fully deactivates β2-adrenergic receptor

**Figure 4:**
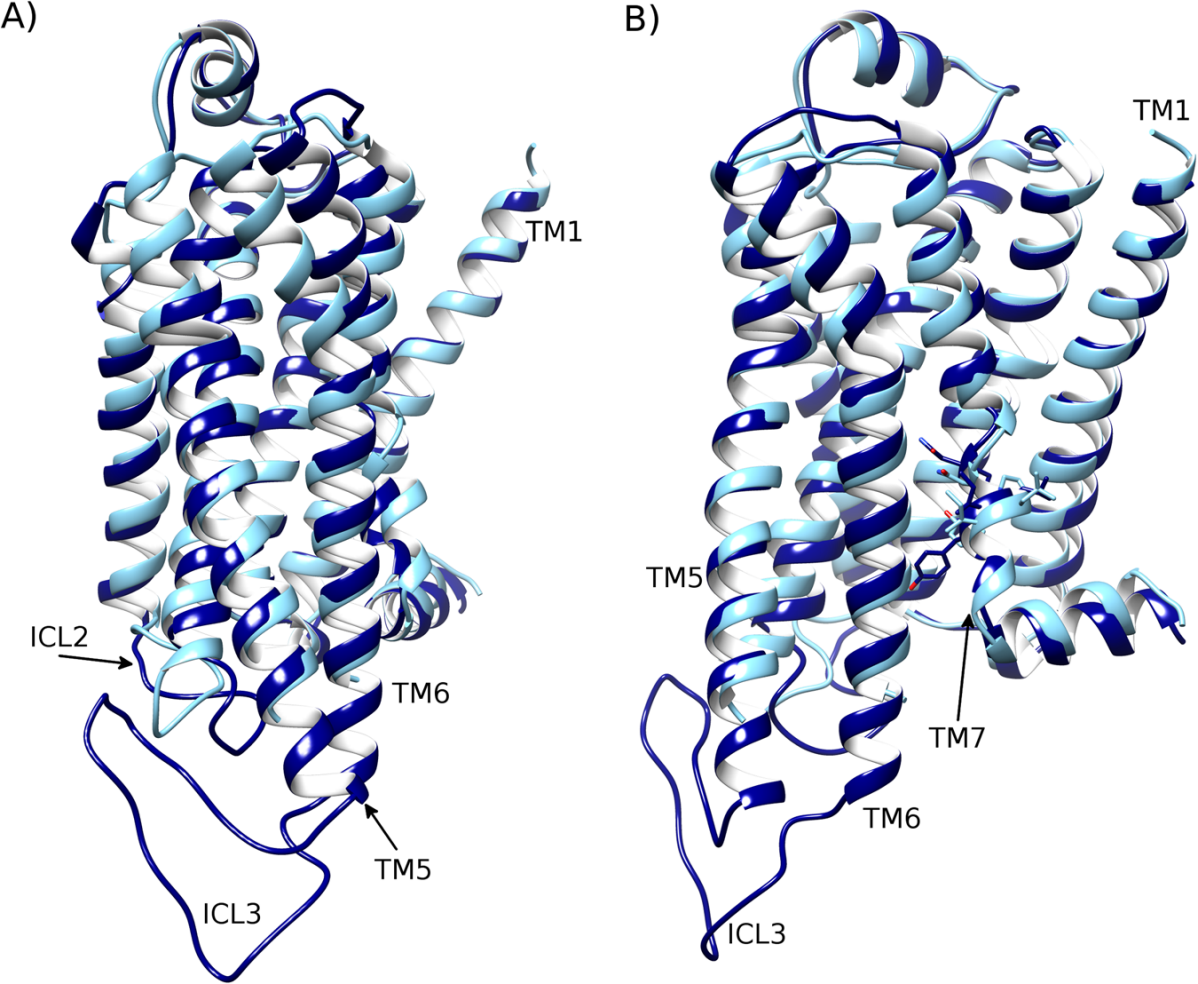
Full deactivation of β_2_AR in a DOPE membrane. Superposition of the inactive crystal structure of β_2_AR (PDB id: 2RH1, light blue) and the conformation of β_2_AR (dark blue) achieved after a 4 μs MD simulation within a DOPE membrane, showing a 90° rotation between (**A**) and (**B**) around the membrane plane (extracellular-side: top, intracellular-side: bottom). Beginning from the active crystal structure (PDB id: 3SN6), the receptor reaches an inactive conformation (dark blue), with an almost identical arrangement with respect to the inactive crystal structure (light blue) of helices TM5, TM6, TM7 (residues of the NPxxY motif are displayed), and disordered intracellular loop 2 (ICL2).

**Figure 5:**
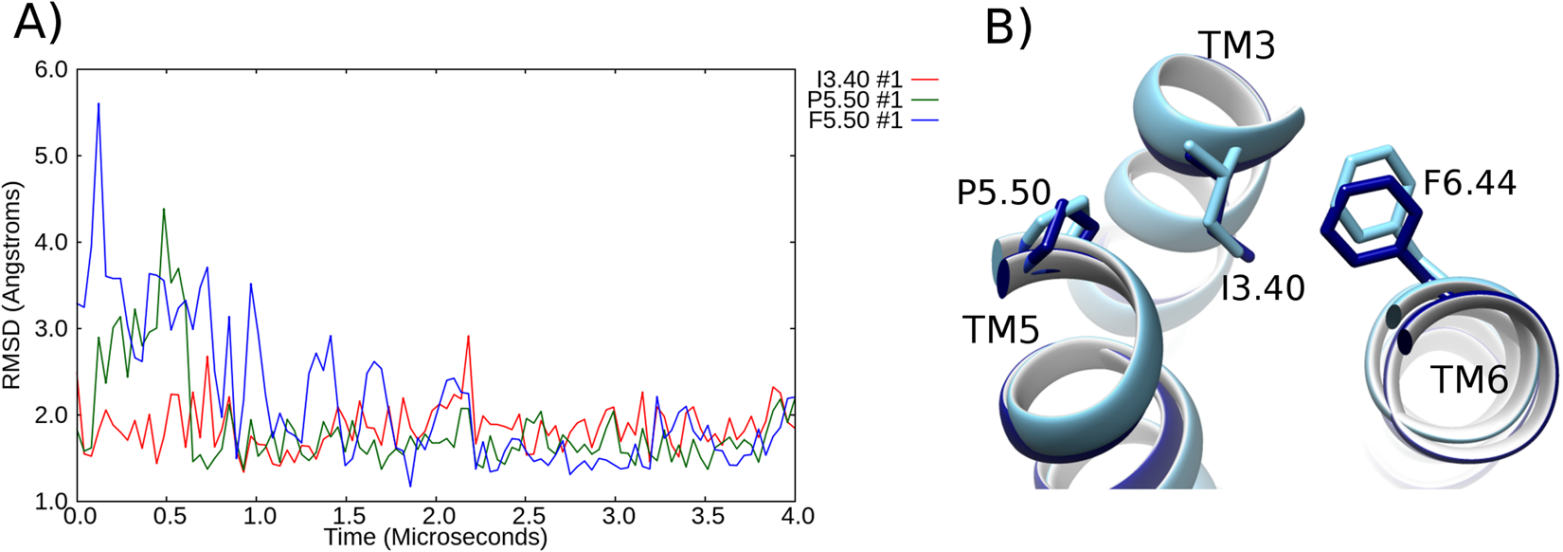
Full deactivation of the triad core of β_2_AR in a DOPE membrane. (**A**) RMSD profile of the conformational changes of the triad core: I^3.40^ (red), P^5.50^ (green) and F^6.44^ (blue) over a 4 μs MD simulation, compared to the inactive crystal structure of β_2_AR (PDB id: 2RH1). In this instance, the MD simulation trajectory, which begins from the active state, is aligned to the inactive crystal structure of β_2_AR (PDB id: 2RH1). (**B**) Superposition of the final triad core conformation achieved after 4 μs (dark blue) against the inactive crystal structure of β_2_AR (light blue). Relevant residues are labelled: proline (P), isoleucine (I), phenylalanine (F) on transmembrane (TM) helices 3, 5, 6.

In a 4 μs MD simulation of β2AR in a homogenous DOPE membrane, like in DOPC, ICL3 shows high flexibility and adopts different conformations during the first 2 μs (SI Fig. 3). During this time, ICL3 does not interact with the membrane but does not reach a stable conformation either. Interestingly, the RMSD profile of TM6 shows faster conformational changes than in DOPC, decreasing from 7.0 Å to 1.9 Å within 1 µs, when comparing with the inactive crystal structure (SI Fig. 4). This suggests an almost instant destabilization of the active state and fast receptor deactivation. Indeed, at 1 μs, TM6 is observed to be in the full inactive conformation (Fig. 4, SI Fig. 4), which does not occur in the DOPC membrane and indicates that DOPE induces greater conformational change, particularly on TM6. In accordance with this inward movement of TM6, the ionic-lock distance also decreases, reaching 7.6 Å between R^3.50^ and E^6.30^ at 1 µs (SI Fig. 5). This is less than the distance of the “open” ionic-lock in the inactive crystal structure (PDB id: 2RH1) and is indicative of a “closed” ionic-lock, a common feature of fully inactive GPCR crystal structures^15^. Regarding the triad core, its RMSD profile reveals an initial perturbation of P^5.50^ and F^6.44^ in the first 0.5 µs and then a rapid packing rearrangement of these residues towards an inactive-like conformation at 1 µs (Fig. 5). Nevertheless, during this time, the receptor continues to have some active-like features on its intracellular side, such as helical ICL2 and shortened TM7. These continue until 2 μs, at which point they also transition into their inactive configurations (Fig. 4, SI Fig. 6). Like in DOPC, ICL3 adopts an inward orientation after 2 μs, interacting with ICL2 (Fig. 4, SI Fig. 3). However, its exact conformation is a little different as it is even more inward than in DOPC, corresponding with what has been previously described as a “very inactive” conformation^15^ and remains mostly stable until the end of the simulation (SI Fig. 3). The receptor finally obtains a full complement of inactive-state features from 2.5 μs onwards, including a stable triad core (Fig. 5), closed ionic-lock (SI Fig. 5), inward TM6 (SI Fig. 4), disordered ICL2 and extended TM7 (Fig. 4), each of which is in agreement with the inactive-state crystal structure (PDB entry: 2RH1)^13^. In addition, each of these features is maintained until the end of the simulation. This suggests that DOPE membranes can induce and maintain deactivation of β2AR. Furthermore, this result has been duplicated in a second MD simulation, revealing the reproducibility of this DOPE-mediated deactivation of β2AR (SI Figs 4–6). Overall, the TM domain transitions from the active state to fully inactive state, including disordered ICL2 and extended TM7 at the NYxxP motif, with an RMSD of 2.5 Å compared to the inactive crystal structure (SI Fig. 6). These observations are in general agreement with the experimental results of Dawaliby *et al*.^44^, who show that DOPE strongly favours the inactive state of β2AR and destabilises the active^44^.

### Lipid DOPG stabilizes active-like state of β2-adrenergic receptor

**Figure 6:**
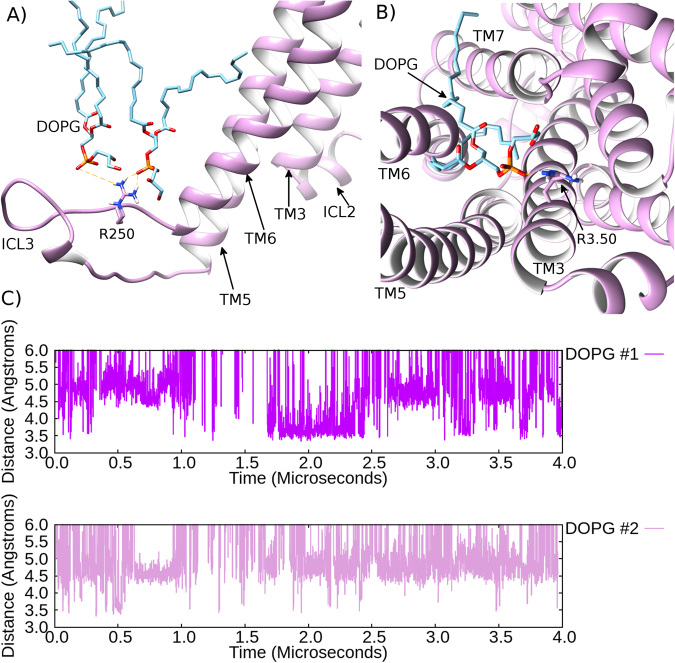
Stabilization of an active-like state of β_2_AR by protein-lipid allosteric interactions. (**A**) An example of DOPG-ICL3/R250 ionic interaction at 4 μs in an MD simulation of β_2_AR (light magenta) within a DOPG (light blue) membrane. (**B**) Intracellular view of β_2_AR (light magenta) where ionic-lock residue R^3.50^ interacts with a DOPG lipid, which protrudes between TM6 and TM7. (**C**) Protein-lipid interactions over time between R^3.50^ and lipid phosphate groups in two MD simulations of β_2_AR embedded in a DOPG membrane (DOPG #1 and DOPG #2, respectively).

**Figure 7:**
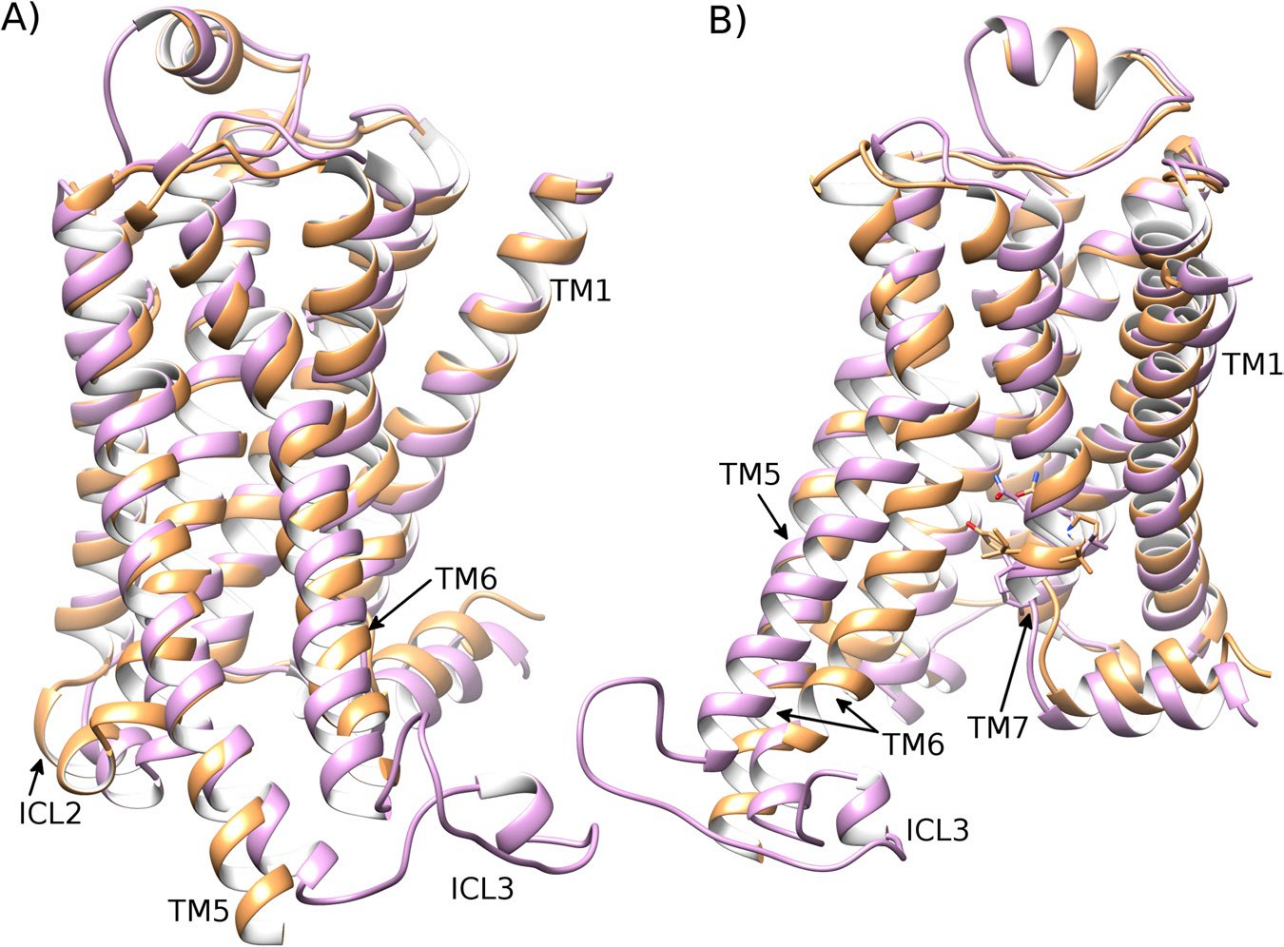
Stabilization of an active-like state of β_2_AR in a DOPG membrane. Comparison of the active-state crystal structure (PDB id: 3SN6) of β_2_AR (orange) and a receptor conformation (light magenta) achieved after 4 μs of an MD simulation within a DOPG membrane, showing 90° rotation between (**A**) and (**B**) around the membrane plane (extracellular-side: top, intracellular-side: bottom). Residues of the NPxxY motif on TM7 are displayed. Due to electrostatic interactions between ICL3/TM6 and the membrane, an active-like state of β_2_AR is stabilized.

**Figure 8:**
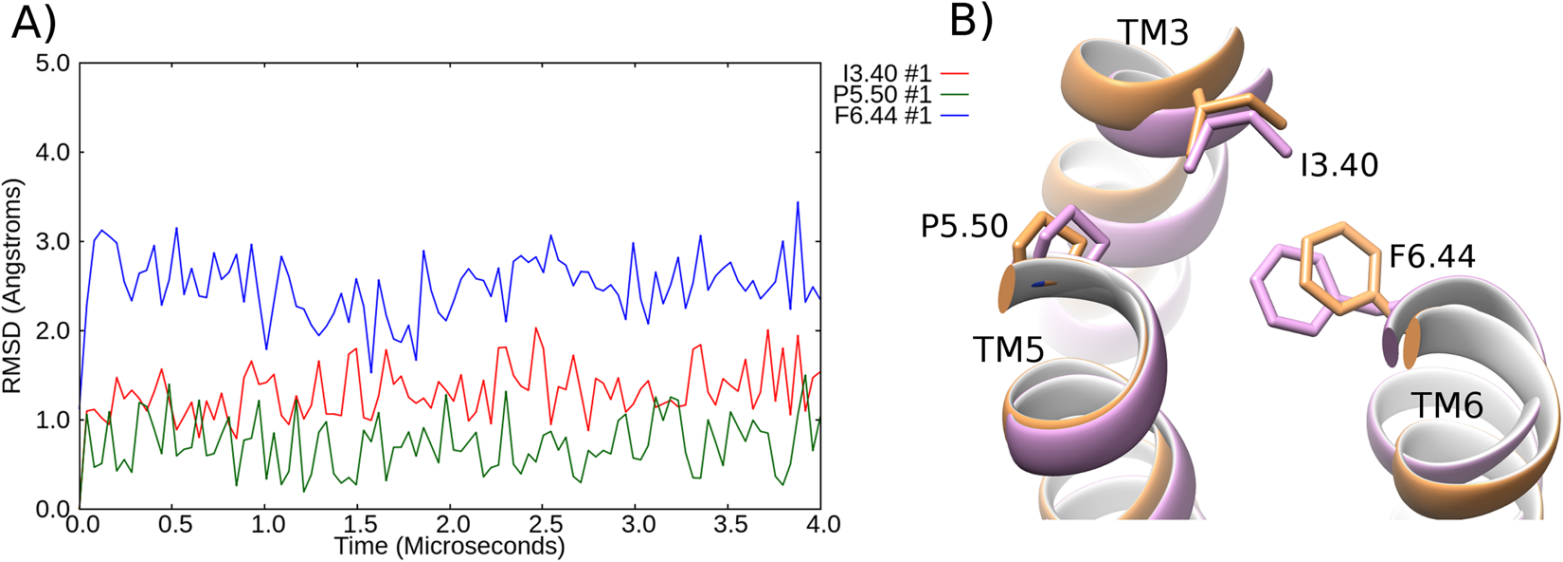
Stabilization of the triad core in an active-like state of β_2_AR in DOPG. **(A**) RMSD profile of the conformational changes of the triad core: I^3.40^ (red), P^5.50^ (green) and F^6.44^ (blue) over a 4 μs MD simulation, compared to the active crystal structure of β_2_AR (PDB id: 3SN6). (**B**) Superposition of the final MD-generated triad core conformation (light magenta) compared to the active crystal structure (PDB id: 3SN6, orange).

In a 4 μs MD simulation of β2AR in a DOPG membrane, there is an initial rapid change in the conformation of ICL3, whose RMSD increases by 15 Å compared to its initial modelled state (in the active crystal structure of β2AR) within the first 0.1 μs (SI Fig. 8A). This is due to ICL3 adopting a more outward orientation (SI Fig. 8B), which begins to see it interacts with the DOPG membrane inner layer (Fig. 6, SI Fig. 7). After 0.3 μs, positively charged residues on ICL3 make close interactions with the neutral headgroups and negatively charged phosphate groups of DOPG lipids (for example, Fig. 6A and SI Fig. 7). These protein-lipid interactions are primarily mediated by R250, and to a lesser extent R253 and H256 (SI Fig. 7). This set of ICL3-DOPG interactions is observed throughout the entirety of 4 µs, exclusively maintaining ICL3 in an outward orientation (with an RMSD of 15–20 Å, SI Fig. 8A). In addition to the influences of ICL3, TM6 appears to be stabilized in its outward conformation by residues located near its N-terminus, such as H269^6.31^, K270^6.32^ and K273^6.35^ (Ballesteros-Weinstein numbering^53^ as superscript, SI Fig. 7), which are observed to interact with phosphate groups of DOPG lipids, presumably providing an outward pull on the helix. Over 4 μs of the simulation, the RMSD of TM6 is 2.0–5.0 Å (compared to the active crystal structure, SI Fig. 9), finishing at 4.3 Å, which is similar to its starting conformation, although slightly more outward (Fig. 7). This may be a consequence of the numerous interactions between ICL3 and TM6 with the membrane, as well as no bound agonist. However, during 1–2 μs, TM6 adopts an almost identical conformation to the active crystal structure with an RMSD of 2.0–3.4 Å (SI Fig. 9), which shows this conformation is also relatively stable. As a result of TM6 and ICL3 maintaining outward orientations, the ionic-lock remains open with a distance of 15–21 Å (SI Fig. 5). This is in agreement with the active-state crystal structure of β2AR^14^. Interestingly, in a previous study, the intrusion of a POPG lipid (a chemically close relative of DOPG, containing an identical lipid headgroup) was observed to occur between TM6 and TM7 of β2AR in its active state^16^. This POPG lipid was specifically observed to interact with R^3.50^ on TM3 (an ionic-lock residue) via a protein-lipid salt-bridge^16^. We can confirm that in addition to the ionic interactions we observe between DOPG lipids and residues on ICL3/TM6, we also observe this extra interaction between R^3.50^ and a DOPG lipid in our MD simulations. Specifically, this interaction is formed between R^3.50^ and the headgroup/phosphate group of a single DOPG lipid bound between TM6 and TM7 at the intracellular side of β2AR (Fig. 6), and appears to assist in maintaining TM6 in an outward orientation. Furthermore, this interaction forms very quickly (within the first few hundred nanoseconds of the simulation) and remains mostly stable over 4 μs, albeit with some fluctuations (Fig. 6). It is also only observed here in a DOPG membrane. Regarding the triad core of β2AR in DOPG, residues I^3.40^, P^5.50^, and F^6.44^ maintain their same rotameric states as observed in the active crystal structure, exhibiting RMSDs of 1.5, 1.0, and 2.4 Å, respectively (Fig. 8).

As a consequence of the previously mentioned protein-lipid interactions and subsequent stabilization of the triad core in an active-like state, the TM domain is observed to remain in an active-like state over 4 μs, including helical ICL2 and shortened TM7 at the NYxxP motif (Fig. 7). This is supported by the overall RMSD of the receptor remaining at 2.0–3.4 Å compared to the initial active crystal structure and finishing at 2.3 Å (SI Fig. 10). Taken together, these results indicate that DOPG lipids are able to stabilize an active-like state of β2AR, even without a bound G protein or bound agonist. These observations are in general agreement with the experimental results of Dawaliby *et al*.^44^, who show that DOPG strongly favours the active state of β2AR compared to DOPE and DOPC lipids. Intriguingly, our results may also offer an explanation of basal activity in β2AR^72^, especially if the lipid environment is chemically similar to that of DOPG.

In order to validate that the DOPG-stabilized receptor conformation was indeed active at a functional level, we re-docked the co-crystallized G_s_α protein (PDB entry: 3SN6) back into the active crystal structure (as a control) and into the MD-generated conformation of β2AR obtained after 4 μs in a DOPG membrane. In both cases, G_s_α is able to adopt an almost identical interaction with the receptor as originally observed in the crystal structure complex without any steric clashes with intracellular loops 1 or 2 (SI Fig. 11). The re-docking of the active crystal structure of β2AR with G_s_α generates an I_sc score of −8.0 (ROSIE Interface score from 0 to −10; more negative is better with −5 a threshold for respectable interaction) while the resulting conformation of β2AR in DOPG with G_s_α has an I_sc of −7.5. This suggests a similar mode of interaction between G_s_α and β2AR embedded in a DOPG membrane as observed in the original active crystal structure. As an additional comparison, we also attempted to dock G_s_α into β2AR conformations generated at the end of respective MD simulations in DOPC and DOPE membranes. In the case of DOPC, a partial interaction between G_s_α and β2AR was possible resulting in an I_sc of −5.8, although G_s_α is positioned at a different angle to the active crystal structure and not fully inserted into the receptor. In the case of DOPE, docking is poor with an I_sc of −4.7 and G_s_α is unable to insert into the receptor, making only superficial contacts (SI Fig. 11).

### Membrane characteristics and lipid headgroups facilitate allosteric modulation of β2AR

**Figure 9:**
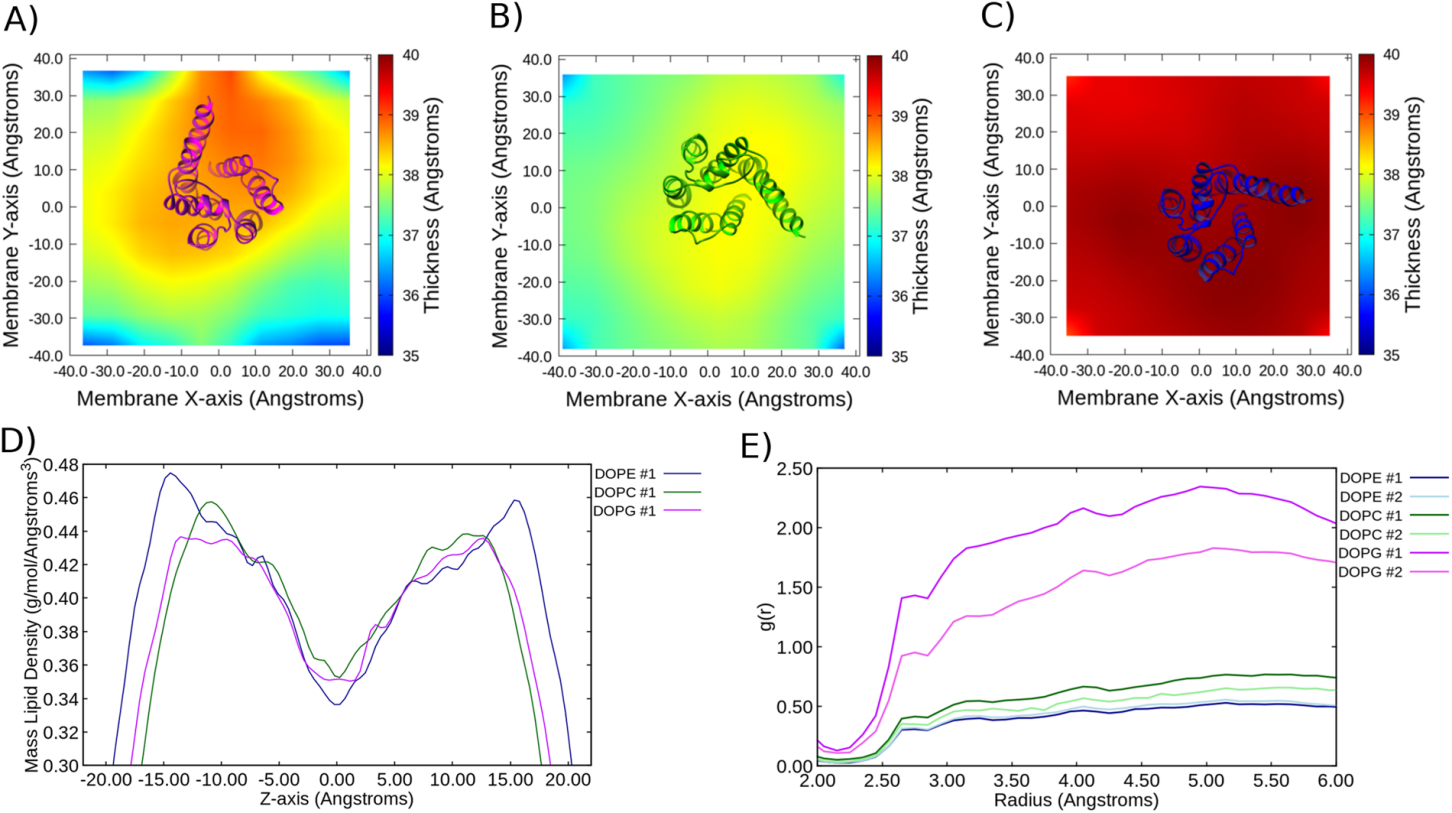
Membrane physical characteristics and effect on β_2_AR conformation. Membrane thickness measurements from 4 μs MD simulations of β_2_AR in (**A**) DOPG, (**B**) DOPC and (**C**) DOPE, respectively. (**D**) Average membrane density measurements from same MD simulations. (**E**) Radial distribution *g(r)* of TM6 with respect to lower leaflet lipids from MD simulations of β_2_AR in three different membranes.

In an effort to explain the different effects that DOPC, DOPE and DOPG membranes have on β2AR conformational change, we sought to quantify the physical characteristics of each membrane and their specific effects on protein-lipid interaction, specifically with regard to the intracellular conformation of TM6 (key for GPCR activation^3–5,20,21^). Time-averaged membrane thickness measurements reveal that DOPG possesses the greatest degree of intra-membrane variation, with >3.0 Å thickness difference between its periphery and core (i.e. thicker around the protein). This compares with an internal variation of <2 Å for DOPC and <1 Å for DOPE (Fig. 9A–C). This suggests that DOPG lipids preferentially cluster around the protein, whilst less so for DOPC lipids and even less for DOPE whose membrane is nearly uniform. In addition, DOPE constitutes the thickest membrane (some 1.5–2.5 Å thicker than DOPC and 0.5–4.0 Å thicker than DOPG). Likewise, time-averaged membrane density measurements show that the DOPE membrane is most dense, particularly at the level of its headgroups (both upper and lower leaflets, Fig. 9D) whilst DOPG and DOPC membranes are less dense. This translates into an average area per lipid of 61 Å^2^ for DOPE, 68 Å^2^ for DOPC and 71 Å^2^ for DOPG, which are consistent with previously calculated theoretical and experimental measurements^73,74^. As intracellular interactions between phospholipids and the receptor appear to facilitate allosteric modulation in DOPG, we calculated the time-averaged radial distribution *g(r)* of TM6 with respect to surrounding lipid phosphate groups in the lower leaflet of each membrane, respectively. This analysis shows that TM6 as a whole in a DOPG membrane has far greater propensity for approaching lipid phosphate groups than in DOPC or DOPE (Fig. 9E). In addition, this propensity is even lower in DOPE than in DOPC, suggesting a different TM6 conformational landscape in each. In order to probe further, we calculated *g(r)* a second time, now for just positively charged sidechains located at the intracellular end of TM6 (four lysine residues: K263^6.25^, K267^6.29^, K270^6.32^, K273^6.35^, see SI Fig. 12). This analysis reveals peaks in *g(r)* at 4.5–5.0 Å in all three membrane types (DOPG > DOPC > DOPE), which likely reflects electrostatic interactions between positive charges on TM6 and negative charges on lipid phosphate groups. To confirm this hypothesis, we calculated the mean number of electrostatic interactions between charged protein and lipid atoms (distance cut-off <4.5 Å) in the lower leaflet of each membrane (SI Fig. 12). Results show that in DOPG, TM6 makes more protein-lipid electrostatic interactions (2.8 atom-pairs ± 1.5 SD) than in either DOPC (1.1 atom-pairs ± 1.0 SD) or DOPE (0.7 atom-pairs ± 0.8 SD). Although these differences may not appear marked, over the course of respective MD simulations their effects are cumulative, leading to considerable differences in TM6-lipid attraction. Taken together, the differences in protein-lipid interactions, TM6 conformational landscapes and physical membrane characteristics, a picture of two sets of forces becomes clear. Firstly, attractive protein-lipid interactions, particularly with respect to TM6, maintain the protein in an active-like state in a DOPG membrane, with these same interactions considerably weaker in DOPC and DOPE membranes, respectively. Secondly, different membrane thickness and densities, particularly between DOPC and DOPE, appear to exert different effects on protein conformation. In the case of a DOPE membrane, its greater density and thickness laterally compresses the protein into an inactive state at a faster rate than in DOPC. On the other hand, in a thinner and less dense DOPC membrane, the protein appears to have greater conformational freedom and inactivates slower. At the heart of these differences lie different lipid headgroups. For example, the positively charged headgroup of DOPE lipids create unfavourable interactions with positively charged residues located on TM6, as well as enabling inter-lipid H-bonds between the headgroup of one lipid with the phosphate group of its neighbour (for example, see SI Fig. 13). These charged inter-lipid interactions contribute to the greater density of a DOPE membrane. On the other hand, the more hydrophobic headgroup of DOPC lipids facilitates moderate interaction with TM6, and an absence of inter-lipid H-bonds contributes to lower membrane density.

## Discussion

Taking profit of recent experimental data, which shows phospholipid-mediated allosteric modulation of β2AR activity^44^, we decided to study the molecular basis behind these effects by simulating β2AR in three different homogeneous membranes of DOPC, DOPE and DOPG using atomistic molecular dynamics. In remarkable agreement with the experimental data^44^, we have been able to identify how DOPC, DOPE and DOPG differentially modulate β2AR activity. This receptor modulation consists of either: partial inactivation, full deactivation or stable activation, respectively, and is seemingly governed by the chemistry of protein-interacting lipid headgroups, as no agonists, antagonists, G proteins or nanobodies are included in any of our MD simulations.

DOPG, with its neutral headgroup and negatively charged phosphate group, appears to play a critical role in the stabilization of the active state of β2AR by making several electrostatic protein-lipid interactions. Firstly, ICL3, through its positively charged residues R250, K253, and R259, interacts with the negatively charged DOPG phosphate groups. Once ICL3 has interacted with the membrane, it is able to maintain a continuous interaction. Secondly, TM6 is able to maintain its outward active conformation through the influences of ICL3, as well as by its own specific attractive interactions between H269^6.31^, K270^6.32^ and K273^6.35^ with DOPG phosphate groups. In addition, the ionic-lock residue R^3.50^ on TM3 is able to interact with the phosphate group of a single DOPG lipid, which is able to protrude between TM6 and TM7 on the intracellular side of the receptor. This appears to assist in the stabilization of the active-like conformation of TM6, although is perhaps less significant than the more direct protein-lipid interactions involving TM6 and ICL3. Consistent with these multiple electrostatic protein-lipid interactions, the radial distribution *g(r)* of TM6 with respect to lower-leaflet DOPG lipids is much more pronounced than in other membranes. These results are notable because it has been suggested previously that the active state of GPCRs, including β2AR, could only be stabilized in MD simulations by bound G proteins or mimetics^3–5,33^. However, our results instead support a pivotal role of lipids in the stable activation of β2AR, even in the absence of a bound agonist or G protein, ensuring the receptor remains in a conformation that is suitable for G protein binding. Although speculative, this might be the case for other homologous GPCRs too. On the contrary, in a DOPC homogeneous membrane, we observe a slow gradual inactivation of β2AR, which reaches an intermediate-like state after 4 μs. This is an expected result because it is in general agreement with other published MD simulations regarding β2AR in phosphatidylcholine (PC) lipid membranes, with POPC being the most commonly employed^4,5,16,18,22–28,30–33^. This supports the notion that the active state of β2AR is inherently unstable and will gradually inactivate without strongly favourable electrostatic protein-lipid interactions (or bound G protein). By analysing the chemical structure and dynamic behaviour observed in DOPC, it is clear why this lipid is unable to make ionic interactions with β2AR. Although DOPC contains a dipole consisting of PO_4_^−^ and N^+^ groups, it has three methyl groups bonded to N^+^, meaning its headgroup is bulky, hydrophobic and tilted parallel to the surface of the membrane as it seeks to minimize contact with water^45^. As a consequence, its headgroup partially obstructs electrostatic interactions between its phosphate group and positively charged residues on the intracellular side of the receptor. Thus, on average we observe fewer TM6-lipid electrostatic interactions and lower TM6 radial distribution *g*(*r*) in a DOPC membrane compared to DOPG. Finally, the DOPE membrane elicits a similar effect to that of DOPC as it also promotes destabilization of the β2AR active state. However, the kinetics of this process is notably quicker. In this case, β2AR undergoes full deactivation to its inactive state within 1.0–2.5 μs (depending on simulation analysed). As DOPE does not contain methyl groups on N^+^, its headgroup is more hydrophilic than DOPC, therefore not as tilted, and instead orientated towards the water phase^45–47^. The exposed N^+^ of DOPE may also have a repulsive effect on positively charged residues located at the intracellular side of TM5, TM6, and ICL3. In particular, this contributes to accelerated deactivation of β2AR by restricting TM6-lipid electrostatic interactions. In addition, as the DOPE membrane has higher density and thickness compared to DOPC, receptor conformation is more restricted with lower TM6 radial distribution *g(r)*.

An additional feature of β2AR-lipid interaction involves the distortion or thickening of the membrane in the vicinity of the receptor. This is particularly noticeable in DOPG, where membrane thickness increases up to 3 Å or more within a radius of ~10 Å around the protein. Interestingly, this effect is also noticeable in DOPC, although to a lesser extent but seems absent in a DOPE membrane. These observations are similar to membrane distortions observed around rhodopsin, where the active state (Meta II) creates local bilayer thickening, not apparent with the inactive state (Meta I)^75^. This can be explained by an increase in the hydrophobic thickness of rhodopsin observed during its activation process, which should then be matched by lipids that are in close proximity^75,76^. Likewise, a similar effect can be seen with sarcoplasmic reticulum Ca^2+^-ATPase, where conformational change between E1 and E2 states alters the hydrophobic mismatch between protein and membrane, resulting in local bulging or pinching of the bilayer^77,78^. Being homologous to rhodopsin, it is consistent that the active state of β2AR creates local thickening of the membrane, such as that observed here in a DOPG membrane, in particular. Also, as the active state of β2AR is destabilized in a DOPC membrane (or fully deactivated in DOPE), it follows that local thickening is less apparent (or absent). Extrapolating from these observations, it would also appear that it is not so much the greater thickness of a DOPE membrane that enhances β2AR deactivation, but rather its pattern of lipid headgroup charges and associated higher membrane density.

As has been described previously, the active and inactive crystal structures of β2AR contain different packing arrangements of their triad core^4,65^. In DOPE membranes, we observe the conformational transition of this triad core from active to inactive. This occurs due to the inward movement and rotation of TM6 towards the core of the receptor. This allows F^6.44^ to move away from P^5.50^ and relocate onto the other side of I^3.40^. However, in DOPC membranes, β2AR does not reach the full inactive state within the simulated time period. As a result, the triad core fluctuates between inactive and active conformations, with F^6.44^ able to adopt both inactive-like and active-like orientations due to an intermediate conformation of TM6, which is neither fully inward nor outward. In addition, I^3.40^ experiences fluctuations in its rotameric state reflecting both inactive-like and active-like conformations. Finally, in DOPG membranes, the triad core of β2AR is seen to maintain an active-like state, albeit with some minor fluctuations in F^6.44^, which is mainly a consequence of initial fluctuations in TM6 (interactions with the membrane are dynamic). Some of these fluctuations in TM6 may also be enhanced by the lack of a bound agonist, which might otherwise help to further stabilize the active state.

**Figure 10:**
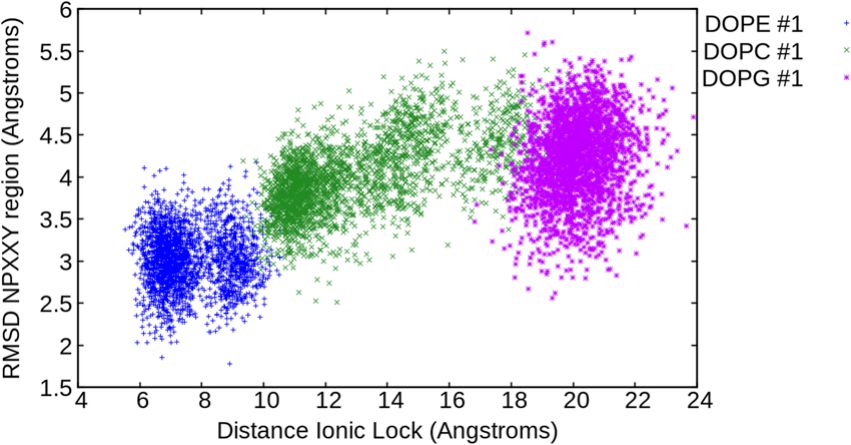
Conformational sampling of β_2_AR in apo state within DOPC, DOPE and DOPG membranes over respective 4 μs MD simulations. Conformational change (RMSD compared to inactive crystal structure, PDB id: 2RH1) of TM7 motif NPxxY against distance between ionic-lock residues (R^3.50^ and E^6.30^). Data is extracted from 2 to 4 μs of each respective MD simulation.

A key factor in our observations of lipid-mediated allosteric modulation of β2AR is the action of ICL3. This non-crystallized region is often neglected in computational studies^4,5,16,18,22,26–28,30–33^ but has been shown to be important by experiments^43^. In our experience, it is critical that this highly flexible loop is included as it provides one of the earliest sources of protein conformational change in our MD simulations, making electrostatic interactions with the DOPG membrane (or indeed not making them in the case of DOPC or DOPE). Although we have not directly tested it, we do not believe the active state of β2AR would be as readily stabilized in DOPG membranes without ICL3, as interactions between this loop and the membrane are so prominent. On this basis, we conclude that ICL3 should ideally be included in all MD simulations of β2AR, as well as that of other GPCRs, regardless of membrane composition. Despite these observations, other conformational changes besides ICL3 are also important. In particular, TM6 is highly influential in the conformational selection of β2AR, and provides a connection between ICL3 and the triad core at the centre of the receptor. In addition, the conformation of TM7, which although not directly connected to the triad core or ICL3, is also important, particularly in defining the state of the intracellular G protein binding-site of β2AR, which displays differences depending on membrane environment. In particular, the conformation of the NPxxY motif^4^ located on TM7 (in terms of RMSD) can be used to precisely define receptor state when combined with ionic-lock distance information between TM3 and TM6 (Fig. 10 and SI Fig. 14). In this schematic, the stabilized active-like state of β2AR in DOPG is clearly distinguishable from that of β2AR in DOPC (intermediate) or DOPE (inactive). As a consequence, a G_s_α protein can be docked into the DOPG-modulated receptor conformation, obtaining an almost identical fit to that observed in the G_s_ protein-bound crystal structure of β2AR^14^. Another intriguing aspect of β2AR (and other GPCRs in general) is its known ability to activate G proteins, albeit at a low level, even without the presence of agonists^72^. This is referred to as basal activity^72^. It is also known that the inactive state of β2AR resides at a lower energy level than the active state^72^. Therefore, in order for the receptor to access and/or remain in the active state, compensating energetic interactions are needed. Our results suggest that in addition to a bound agonist (which is not explicitly simulated here) anionic lipids may provide this additional energy through interactions with the protein, allowing β2AR to reach its active state. Indeed, this suggests a role of lipids in the regulation of β2AR basal activity. Likewise, the role of cationic lipids, especially those with an exposed ammonium group, may be to assist in the deactivation of β2AR, thereby reducing basal activity.

Although beyond the scope of this study, rather than stabilizing or destabilizing the active state of β2AR, it is interesting to speculate if the reverse process of activating the inactive state of β2AR could also be induced by lipids during MD simulations. To our knowledge this has not yet been achieved. However, we believe it could be theoretically possible, although longer simulation times than those performed here may be required. In addition, a bound agonist may also be required to accelerate the kinetics of the process. It is certainly an interesting prospect, and if obtainable, could confirm the hypothesis that lipids have a stronger effect on GPCRs than previously thought. It may also be possible to directly demonstrate basal GPCR activity, for which MD simulations have not yet been able to satisfactorily model. Intriguingly, it has yet to be determined experimentally whether other GPCRs are sensitive to phospholipids in the same way as β2AR. From our computational results, we observe that electronegative lipids strongly act on β2AR to stabilize its active conformation(s), while lipids with positively charged headgroups may act to deactivate it. This may hold true for other homologous GPCRs that possess similar patterns of positively charged residues on TM6 and ICL3. Another interesting question concerns how heterogeneous membranes, which likely reflect mammalian physiology more accurately than homogenous membranes, might affect GPCR behaviour through different blends of phospholipids containing a variety of headgroups and fatty acid chains. This is a complex problem requiring further studies but could reveal how specific cellular environments might differentially regulate GPCR-mediated signalling at the molecular level.

### Associated Content

#### Supporting Information

Comparison of inactive and active β2AR crystal structures; RMSD plots of ICL3, TM6, and whole protein in DOPG, DOPE, DOPC membranes; plot of ionic-lock distances in DOPG, DOPE, DOPC membranes; visual of selected protein-lipid interactions in DOPG membrane; visual of G protein dockings, plots of electrostatic TM6-lipid interactions and radial distribution *g(r)*.

## Electronic supplementary material

Supplementary Information
