# Supplementary material for: Structural insights into positive and negative allosteric regulation of a G protein-coupled receptor through protein-lipid interactions
Source: Sci Rep. 2018 Mar 13;8:4456. doi: 10.1038/s41598-018-22735-6 (PMC5849739; doi:10.1038/s41598-018-22735-6)
Supplement: Supplementary file 1 — Supplementary Information [file 41598_2018_22735_MOESM1_ESM.pdf]

**SUPPLEMENTARY INFORMATION**

# Structural insights into positive and negative allosteric regulation of a G protein-coupled receptor through protein-lipid interactions

Agustin Bruzzese[a], Carles Gil[b], James A.R. Dalton[a,]*, Jesús Giraldo[a,]*.

[a]Laboratory of Molecular Neuropharmacology and Bioinformatics, Institut de Neurociències and Unitat de Bioestadística, Universitat Autònoma de Barcelona, Spain; Network Biomedical Research Centre on Mental Health (CIBERSAM), Spain.

[b]Department of Biochemistry and Molecular Biology, Institut de Neurociències, Universitat Autònoma de Barcelona, Spain

* Corresponding authors: james.dalton@uab.es and jesus.giraldo@uab.es

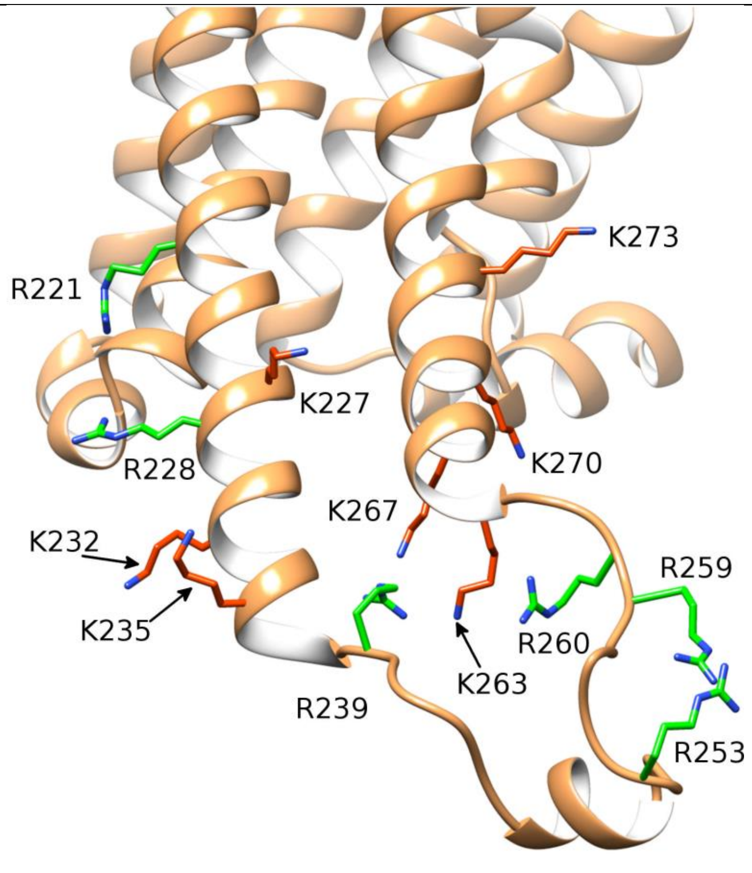


**SI Figure 1. A cluster of positively-charged residues (13 in total) located on TM5, TM6 and ICL3 on the intracellular side of β2AR.** Arginines are coloured green and lysines coloured orange.

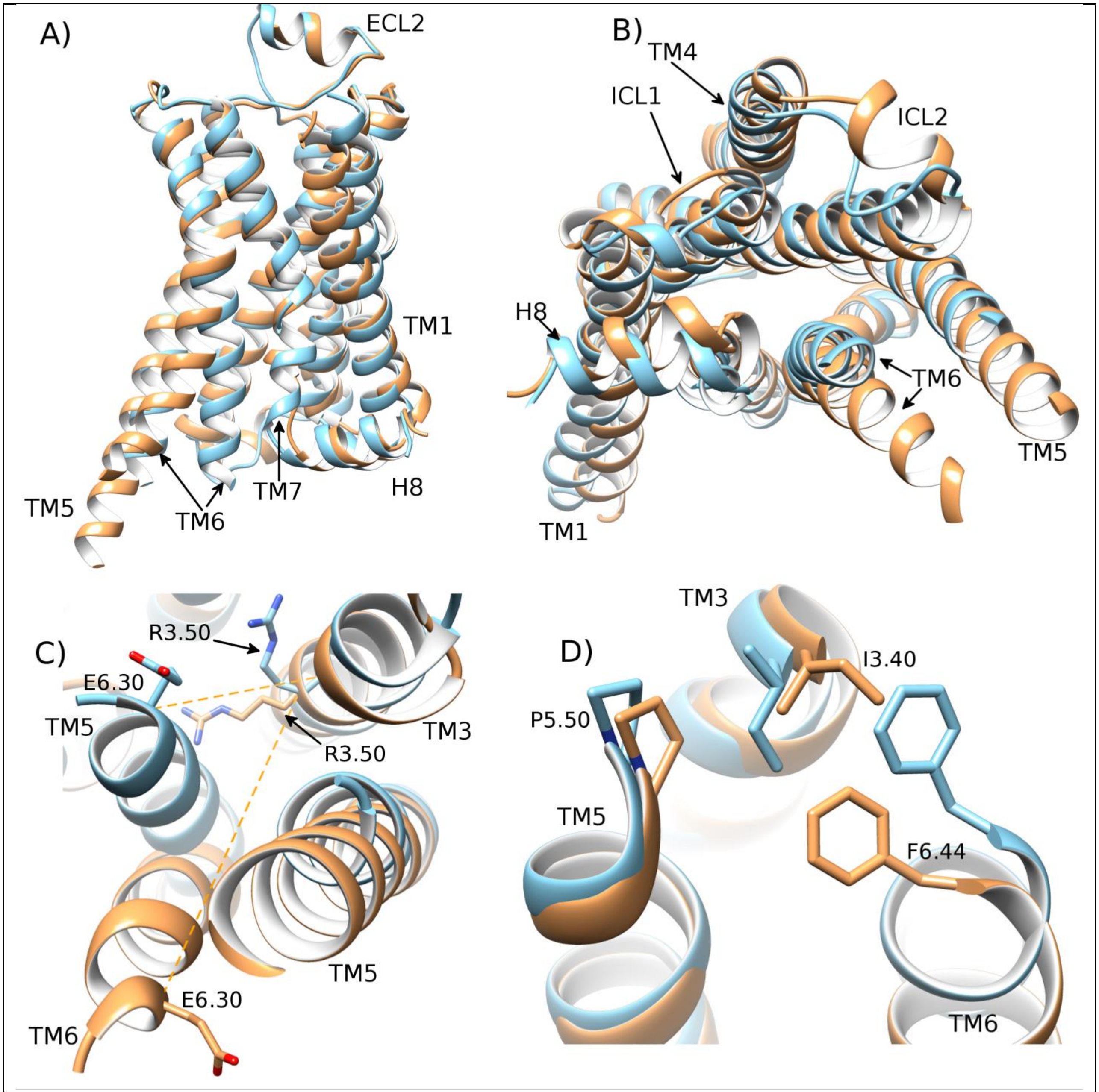


**SI Figure 2. Structural comparison between β$_2$AR active and inactive crystal structures.** A) Structural superposition of the active-state crystal structure (PDB id: 3SN6, orange) on the inactive-state crystal structure (PDB id: 2RH1, cyan). B) Intracellular view of TM6 movement between inactive and active states. C) The intracellular-side of the receptor and the distance (indicated by dashed lines) between ionic-lock residues ($R^{3.50}$ and $E^{6.30}$) in both states. D) Packing of the triad core in active and inactive crystal structures. Relevant structural features are labelled: intracellular loops (ICLs) and transmembrane (TM) helices.

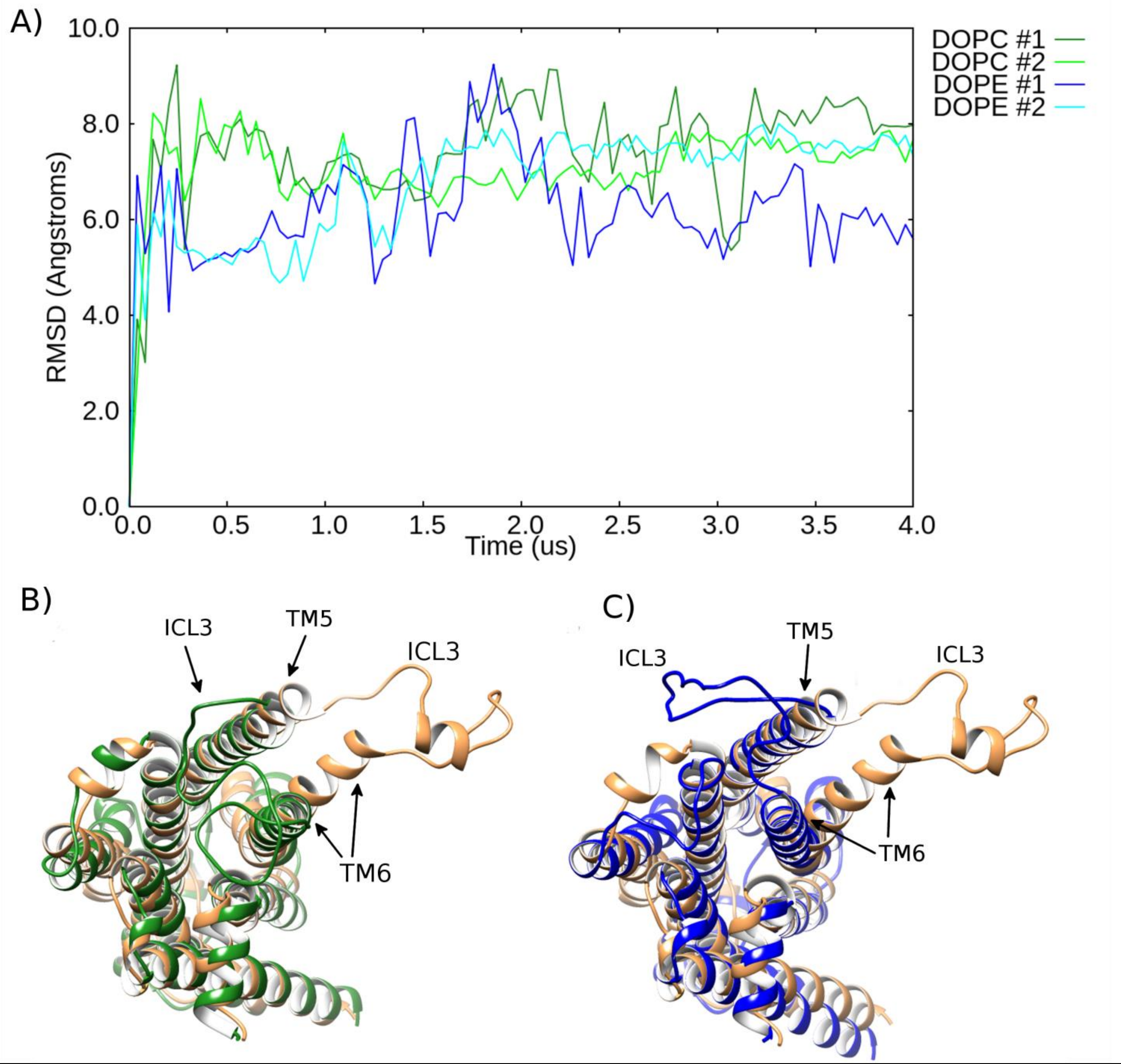


**SI Figure 3. Comparison between the initial conformation of ICL3 and those generated during MD simulations of $\beta_2$AR in DOPC and DOPE membranes.** A) RMSD profiles of ICL3 over respective 4 µs MD simulations compared to the initial modelled conformation. B) Initial active-state conformation of $\beta_2$AR/ICL3 (orange) and the conformation generated after 0.4 µs of MD simulation (dark green) in a DOPC membrane. C) Initial active-state conformation of $\beta_2$AR/ICL3 (orange) and the conformation generated after 2 µs (dark blue) in a DOPE membrane. Intracellular loop 3 (ICL3) and transmembrane (TM) helices 5 and 6 are labelled.

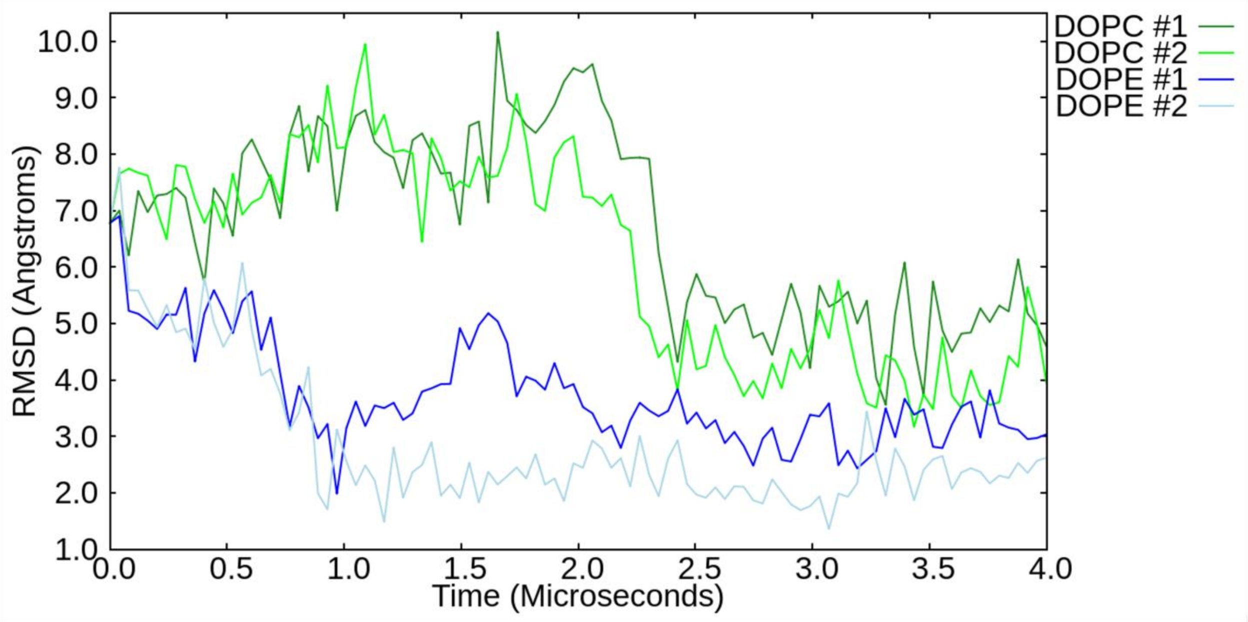


**SI Figure 4. Comparison of the modulation of TM6 conformation in $\beta_2$AR by different lipid membranes.** RMSD profiles of conformational changes in TM6 observed during respective 4 µs MD simulations, compared to the inactive crystal structure of $\beta_2$AR (PDB id: 2RH1). The four lines constitute two MD simulations in DOPC membrane (dark green and light green) and two MD simulations in DOPE membrane (dark blue and light blue).

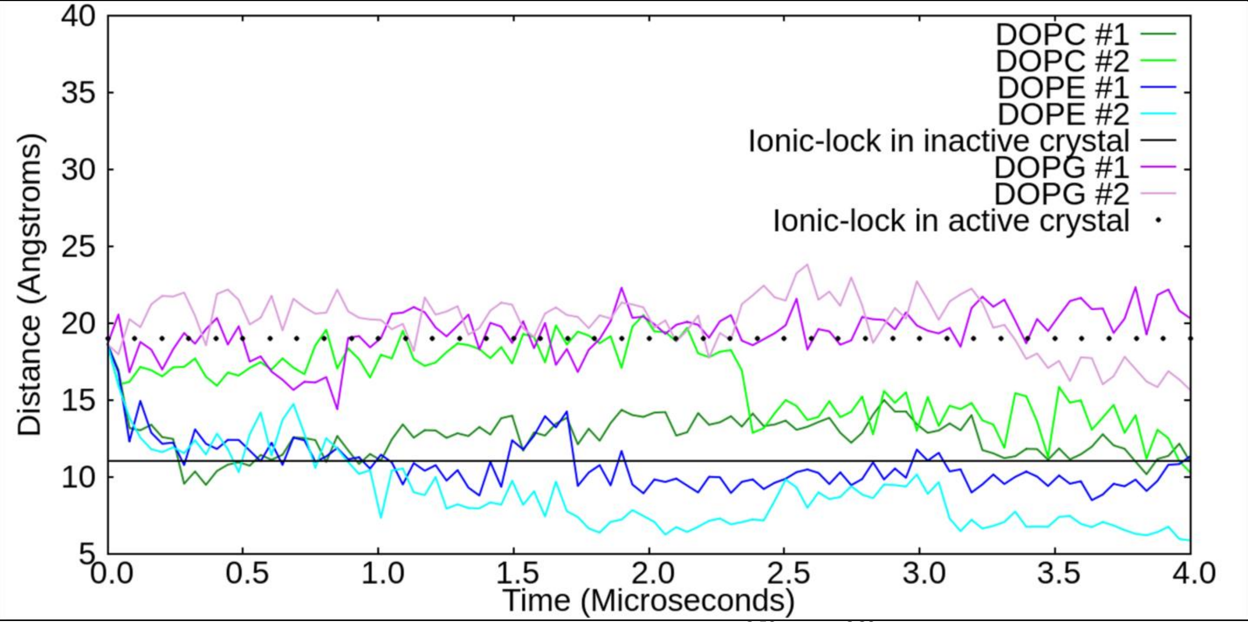


**SI Figure 5. Comparison of distance between ionic-lock residues ($R^{3.50}$ and $E^{6.30}$) as a measure of deactivation or stabilization of the active state of β2AR**. The six oscillating lines show ionic-lock status over respective 4 µs MD simulations of β2AR in DOPC (dark green and light green), DOPE (dark blue and light blue), and DOPG (dark magenta and light magenta) membranes. Corresponding flat-lines are included to show the observed distance in the active (dotted line, PDB id: 3SN6) and inactive (solid line, PDB id: 2RH1) β2AR crystal structures.

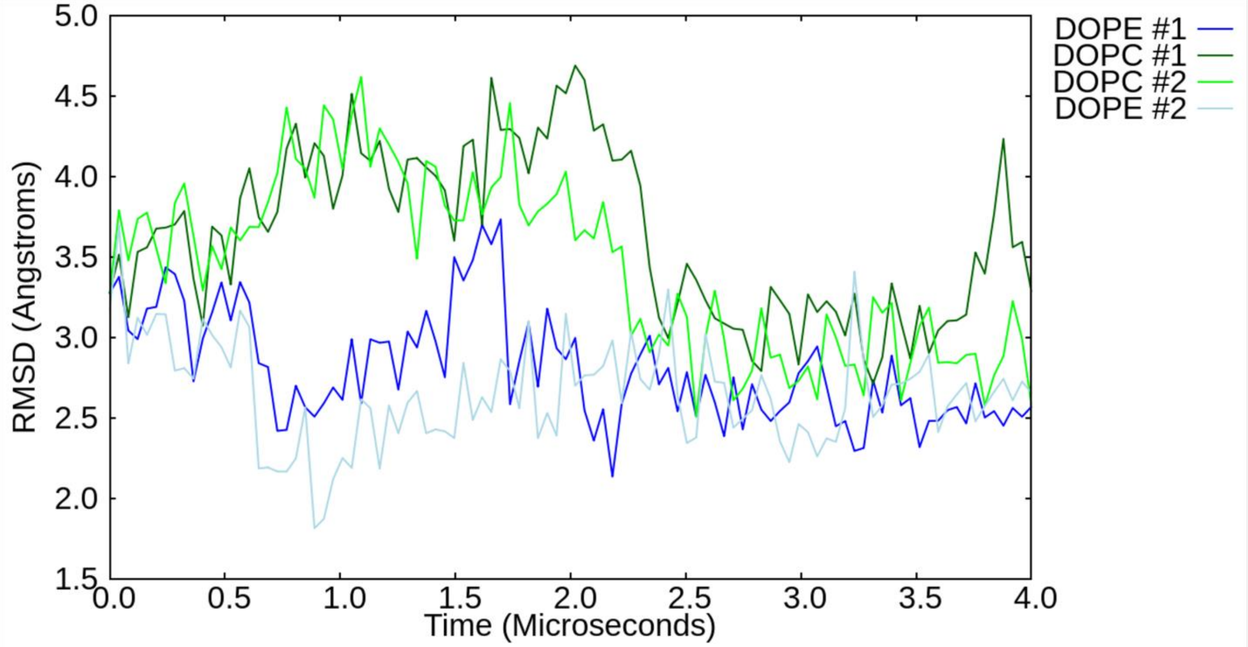


**SI Figure 6. Comparison of full or partial inactivation processes of $\beta_2$AR in DOPE or DOPC membranes.** RMSD profiles of conformational changes in helices 1-8 observed during respective 4 µs MD simulations compared to the inactive crystal structure of $\beta_2$AR (PDB id: 2RH1). The four lines constitute two MD simulations in DOPC membrane (dark green and light green) and two MD simulations in DOPE membrane (dark blue and light blue).

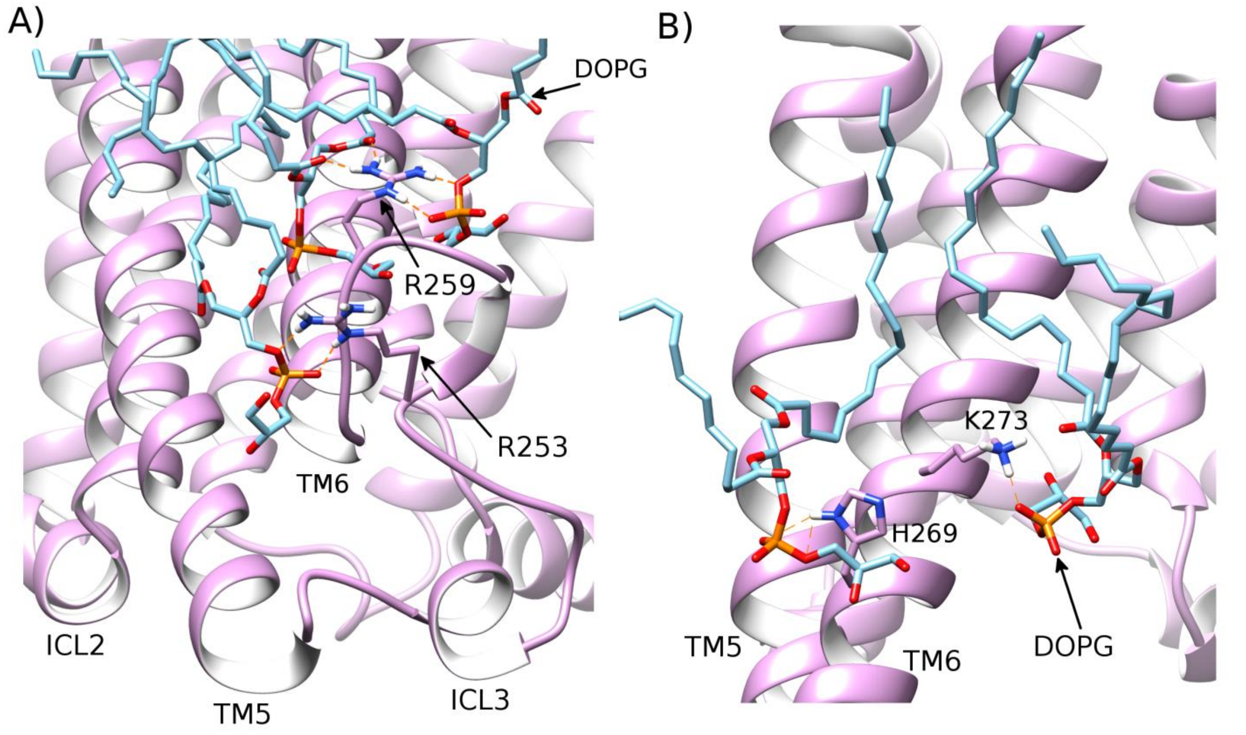


**SI Figure 7. Selected protein-lipid interactions assisting the stabilization of an active-like state of $\beta_2$AR in a DOPG membrane.** A) Selected allosteric interactions between positively charged residues (R253, R259) on intracellular loop 3 (ICL3) of $\beta_2$AR (light magenta) with DOPG lipids (light blue) after 4.0 µs MD simulation. B) Interactions between selected residues on TM6 ($H269^{6.31}$, $K273^{6.35}$) and phosphate groups of DOPG lipids (light blue) allow the stabilization of TM6 in an outward conformation.

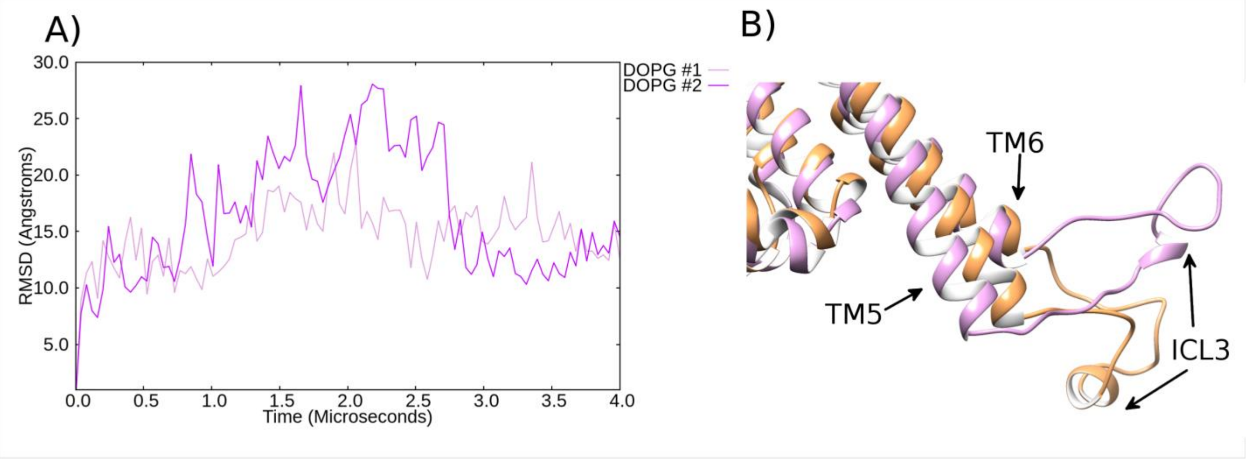


**SI Figure 8. Comparison between the initial conformation of ICL3 and that generated during MD simulations of $\beta_2$AR in a DOPG membrane.** A) RMSD profiles of ICL3 observed during 4 µs MD simulations compared to the initial active-state conformation. B) Initial active-state conformation of $\beta_2$AR (orange) superimposed with the conformation of ICL3 observed after 0.1 µs (light magenta) in a DOPG membrane.

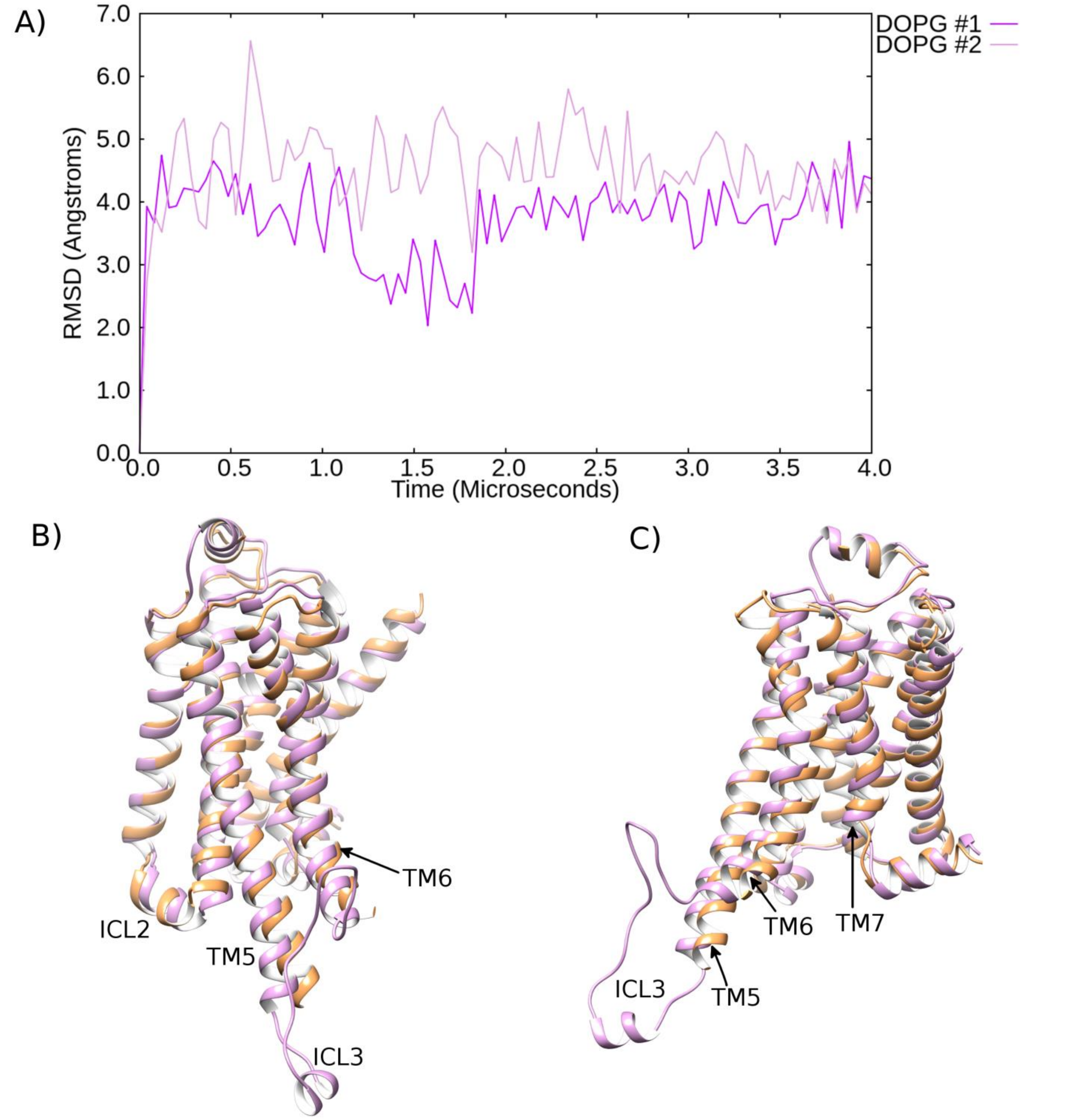


**SI Figure 9. Conformational changes in TM6 of apo β$_2$AR within a DOPG membrane, observed over respective 4 µs MD simulations.** (A) RMSD profiles of the conformational changes of TM6 in two MD simulations (light and dark magenta) compared to the active-state crystal structure (PDB id: 3SN6). (B) and (C) Structural comparison of the active-state crystal structure of β$_2$AR (orange) and a receptor conformation (light magenta) obtained from halfway of its 4 µs MD simulation within a DOPG membrane, showing 90° rotation around the membrane plane (extracellular-side: top, intracellular-side: bottom). Intracellular loops (ICL) 2 and 3, and transmembrane (TM) helices 5, 6, and 7 are labelled.

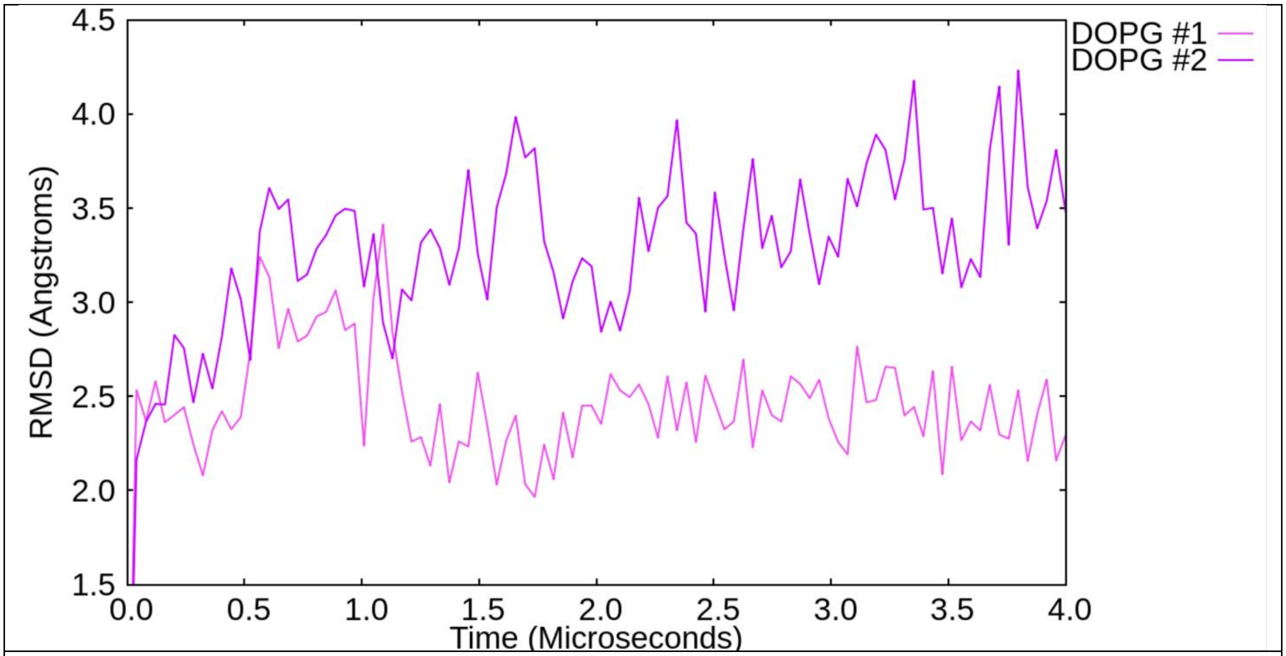


**SI Figure 10. Conformational changes of $\beta_2$AR in apo state within a DOPG membrane, observed over respective 4 μs MD simulations.** RMSD profiles of the transmembrane domain (helices 1-8) compared to the active-state crystal structure (PDB id: 3SN6).

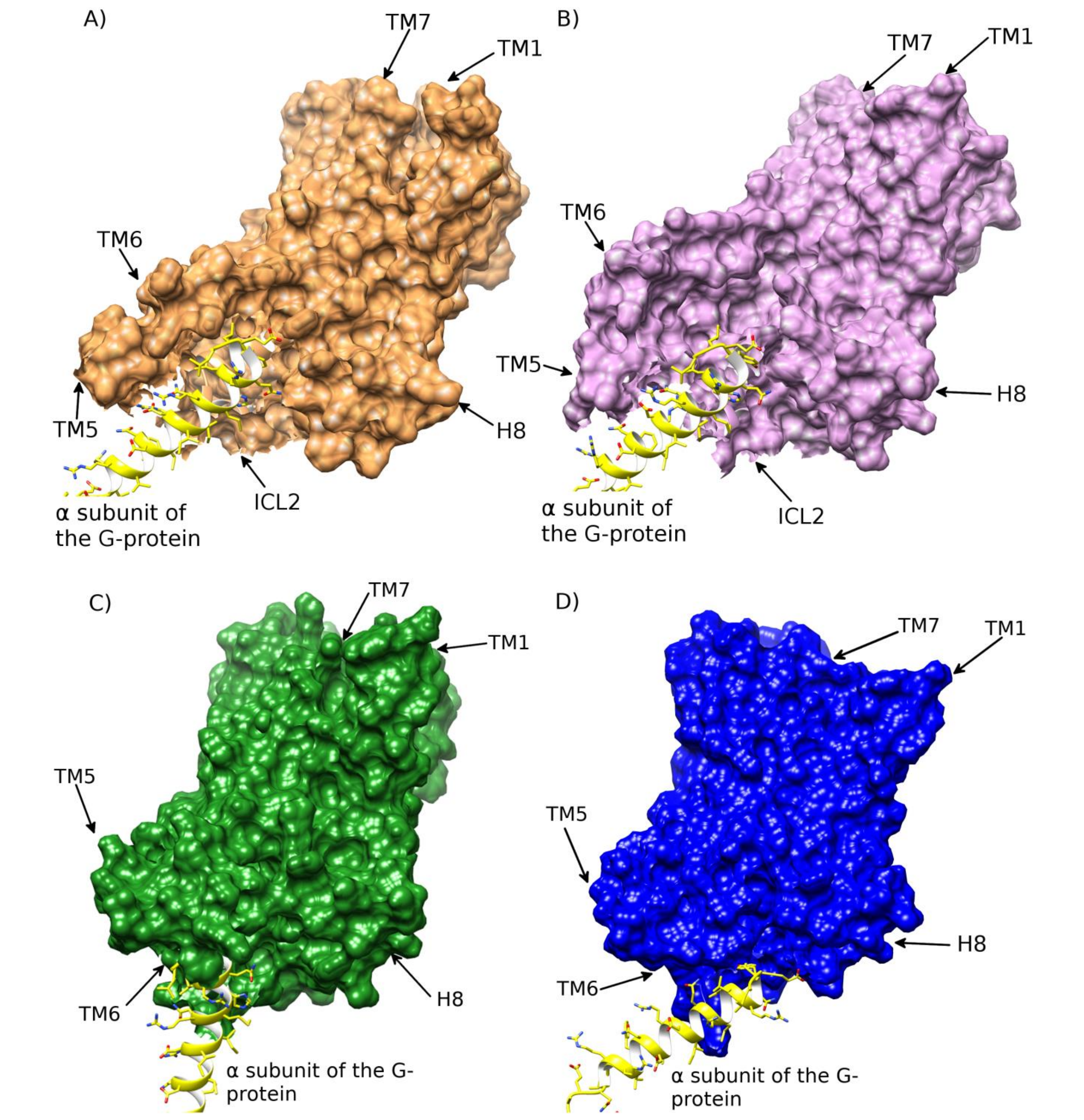


**SI Figure 11. The MD-generated receptor conformation of $\beta_2$AR in a DOPG membrane is able to bind co-crystallized $G_s$-alpha protein similar to the active-state crystal structure (PDB id: 3SN6).** A) Crystal structure of the active state of $\beta_2$AR (PDB id: 3SN6, orange) bound to the C-terminus of its co-crystallized $G_s$-alpha protein (yellow). B) Stabilized active-like conformation of $\beta_2$AR in a DOPG membrane after 4 μs MD simulation (light magenta) is able to correctly dock $G_s$-alpha protein (yellow). (C) Partial inactive conformation of $\beta_2$AR in a DOPC membrane (green) and (D) full inactive conformation of $\beta_2$AR in a DOPE membrane (blue) after respective 4 μs MD simulations are unable to correctly dock $G_s$-alpha protein (yellow).

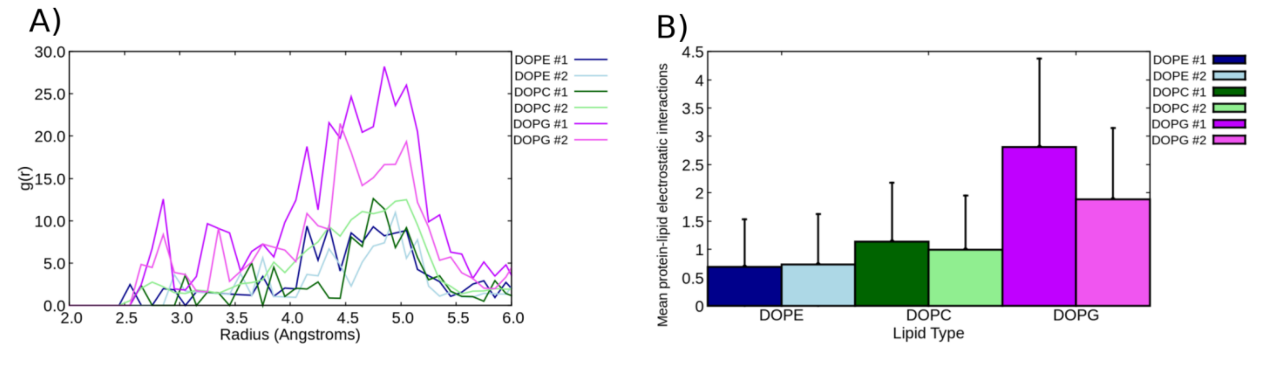


**SI Figure 12. Protein-lipid electrostatic interactions between TM6 of $\beta_2$AR and membrane lower-leaflet.** A) Radial distribution *g(r)* of positively charged residues at the intracellular end of TM6 (K263, K267, K270, K273) and negatively charged phosphate groups of lower-leaflet lipid molecules in three different membranes. (B) Comparison of average observed protein-lipid electrostatic interactions between TM6 of $\beta_2$AR and membrane lower-leaflet over respective 4 µs MD simulations. Error bars represent standard deviation.

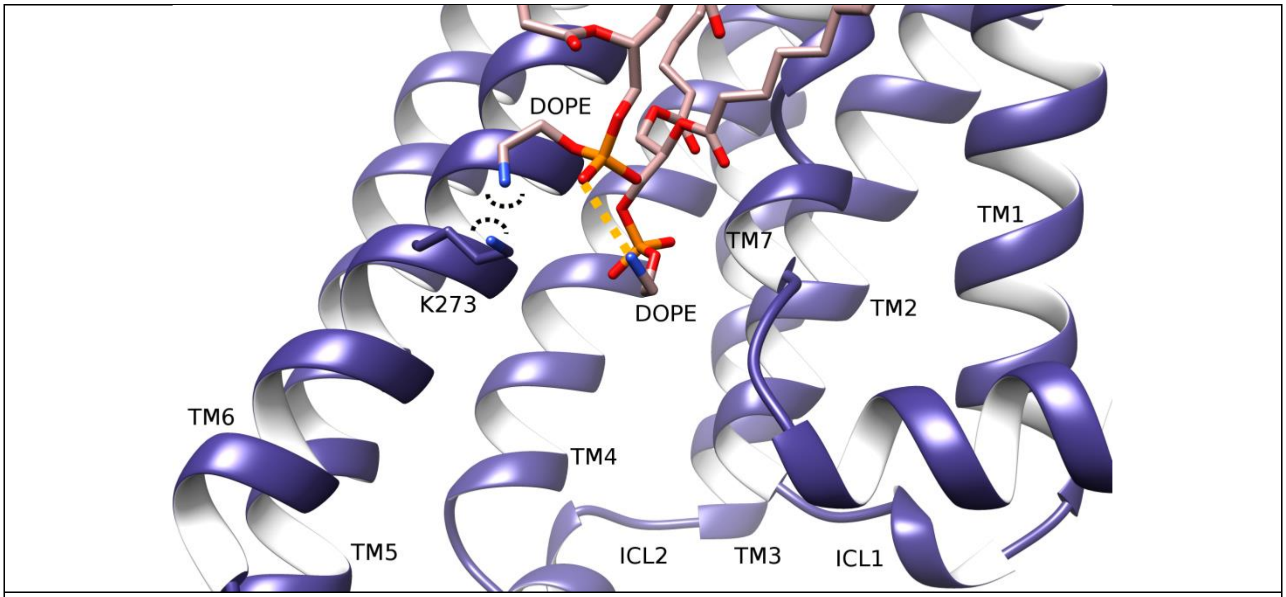


**SI Figure 13. Selected protein-lipid interactions assisting in the deactivation of $\beta_2$AR in a DOPE membrane.** Repulsive allosteric interaction (represented by dotted curved black lines) between K273$^{6.35}$ on TM6 of $\beta_2$AR (dark blue) and positively-charged head-group of DOPE lipid (light brown) during the first nanosecond of its MD simulation. An inter-lipid hydrogen bond is formed between adjacent DOPE lipids (dotted orange line).

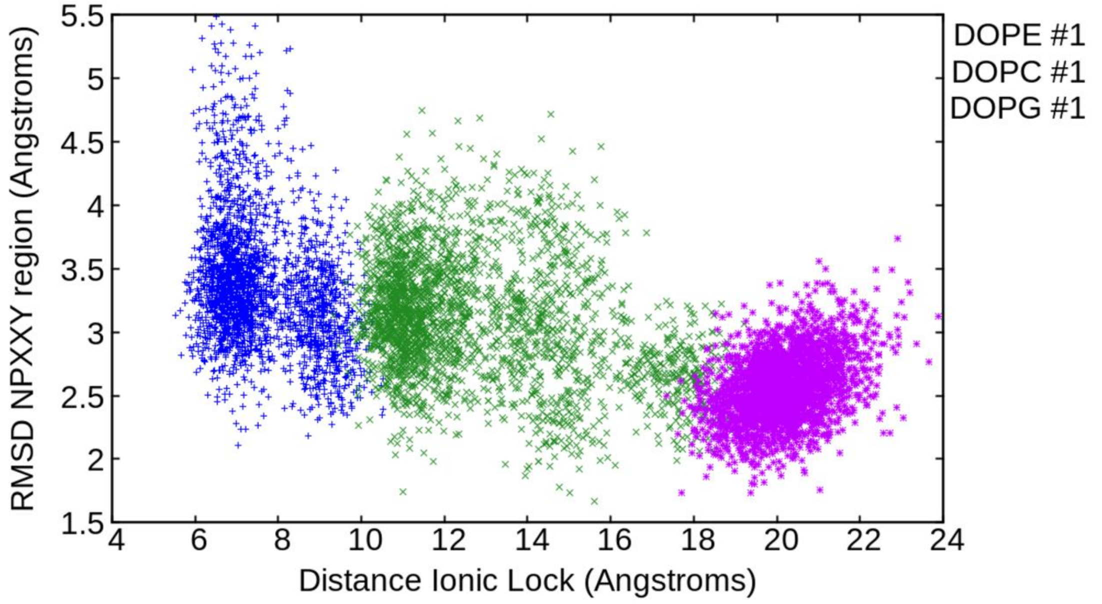


**SI Figure 14. Conformational sampling of $\beta_2$AR in apo state within DOPC, DOPE and DOPG membranes over respective 4 µs MD simulations.** Conformational change (RMSD compared to active crystal structure, PDB id: 3SN6) of TM7 motif NPXXY against distance between ionic-lock residues ($R^{3.50}$ and $E^{6.30}$). Data is extracted from 2 to 4 µs of each respective MD simulation.

**Table S1. Protein conformational state measurements of $\beta_2$AR taken across respective MD simulations in three different phospholipid membranes (DOPC, DOPE, DOPG).**

| | | DOPE | | DOPC | | DOPG | |
|---|---|---|---|---|---|---|---|
| Measure (Å) | Time (μs) | #1 | #2 | #1 | #2 | #1 | #2 |
| Ionic Lock Distance | 0.0 | 18.6 | 18.6 | 18.6 | 18.6 | 18.6 | 18.6 |
| | 1.0 | 11.3 | 7.6 | 11.7 | 17.6 | 18.1 | 21.0 |
| | 2.0 | 9.2 | 7.4 | 14.1 | 19.2 | 19.1 | 21.5 |
| | 3.0 | 10.9 | 8.9 | 13.8 | 14.4 | 19.7 | 21.2 |
| | 4.0 | 11.1 | 5.8 | 11.1 | 10.2 | 20.3 | 16.8 |
| RMSD Transmembrane helix 6 w/t inactive crystal | 0.0 | 6.7 | 6.7 | 6.7 | 6.7 | 6.7 | 6.7 |
| | 1.0 | 2.8 | 2.7 | 8.2 | 8.3 | 7.7 | 8.3 |
| | 2.0 | 3.3 | 2.8 | 9.3 | 8.4 | 8.8 | 9.3 |
| | 3.0 | 3.2 | 1.9 | 4.9 | 4.8 | 8.3 | 8.3 |
| | 4.0 | 3.0 | 2.6 | 4.7 | 4.0 | 8.3 | 7.8 |
| RMSD Transmembrane helix 6 w/t active crystal | 0.0 | 0.0 | 0.0 | 0.0 | 0.0 | 0 | 0 |
| | 1.0 | 5.4 | 5.8 | 6.1 | 2.9 | 3.2 | 4.9 |
| | 2.0 | 6.5 | 6.2 | 3.7 | 3.2 | 3.4 | 4.5 |
| | 3.0 | 5.3 | 6.1 | 4.7 | 3.5 | 3.7 | 4.4 |
| | 4.0 | 5.2 | 6.7 | 4.8 | 4.4 | 4.3 | 4.1 |
| RMSD Helices 1-8 w/t inactive crystal | 0.0 | 3.3 | 3.3 | 3.3 | 3.3 | 3.3 | 3.3 |
| | 1.0 | 2.6 | 2.3 | 4.1 | 3.9 | 4.8 | 4.2 |
| | 2.0 | 2.9 | 2.6 | 4.7 | 3.8 | 4.9 | 4.8 |
| | 3.0 | 2.7 | 2.4 | 3.3 | 2.9 | 4.5 | 4.3 |
| | 4.0 | 2.5 | 2.7 | 3.4 | 2.8 | 4.4 | 4.1 |
| RMSD Helices 1-8 w/t active crystal | 0.0 | 0.0 | 0.0 | 0.0 | 0.0 | 0.0 | 0.0 |
| | 1.0 | 3.9 | 4.2 | 3.9 | 4.7 | 2.3 | 3.1 |
| | 2.0 | 4.5 | 5.0 | 3.9 | 4.4 | 2.4 | 3.0 |
| | 3.0 | 3.5 | 4.9 | 3.3 | 4.7 | 2.3 | 3.2 |
| | 4.0 | 4.8 | 5.0 | 4.3 | 4.4 | 2.3 | 3.5 |
| RMSD NPxxY w/t inactive crystal | 0.0 | 3.7 | 3.7 | 3.7 | 3.7 | 3.7 | 3.7 |
| | 1.0 | 3.2 | 2.9 | 3.2 | 3.0 | 2.8 | 4.2 |
| | 2.0 | 3.1 | 3.0 | 3.5 | 4.7 | 4.6 | 4.2 |
| | 3.0 | 2,8 | 3.0 | 3.9 | 3.6 | 4.7 | 4.9 |
| | 4.0 | 2.6 | 2.7 | 3.6 | 4.0 | 4.5 | 4.8 |
| RMSD NPxxY w/t active crystal | 0.0 | 0.0 | 0.0 | 0.0 | 0.0 | 0.0 | 0.0 |
| | 1.0 | 3.3 | 4.1 | 3.1 | 3.0 | 3.0 | 3.2 |
| | 2.0 | 3.2 | 3.3 | 3.0 | 2.6 | 2.8 | 3.6 |
| | 3.0 | 2.9 | 4.1 | 3.2 | 3.3 | 2.7 | 3.1 |
| | 4.0 | 3.0 | 4.2 | 2.8 | 2.2 | 2.4 | 2.8 |
